# Supplementary material for: Structural and Functional Differences in Small Intestinal and Fecal Microbiota: 16S rRNA Gene Investigation in Rats
Source: Microorganisms. 2024 Aug 25;12(9):1764. doi: 10.3390/microorganisms12091764 (PMC11434385; doi:10.3390/microorganisms12091764)
Supplement: Supplementary file 1 [file microorganisms-12-01764-s001.zip › Figure S3-S4.pdf]

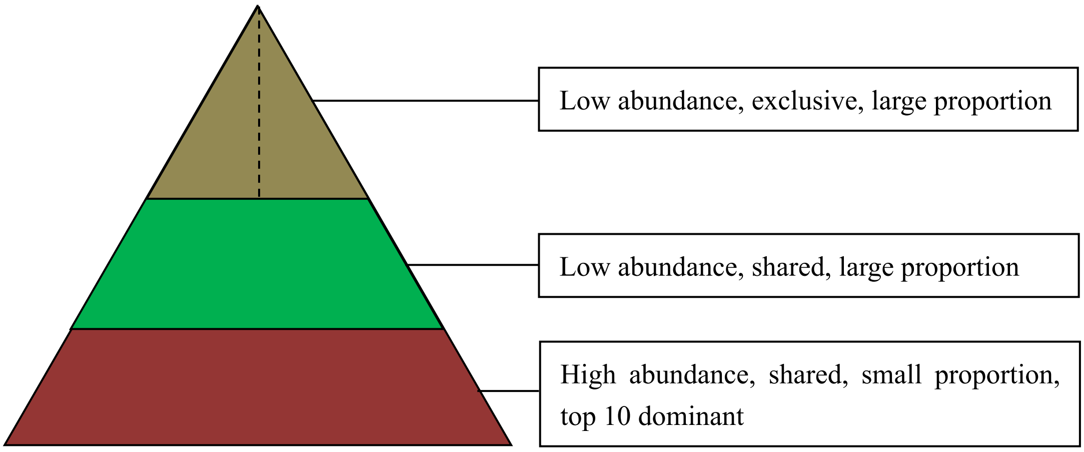

Figure S3. Microecological features of the gut microbiome. Different enteral nutrition environments throughout the gut leads to different compositions of intestinal flora, which in turn leads to different intestinal microecologies. The microbiota of different parts of the intestine have similar microecological characteristics, including dominant and less dominant bacteria. The dominant bacteria are a very small proportion of the total number of species, consisting of the top 10 bacteria contributing 70–80% of abundance. The less dominant bacteria are a large proportion of the total number of species and shared or exclusive between different segments of the intestine.

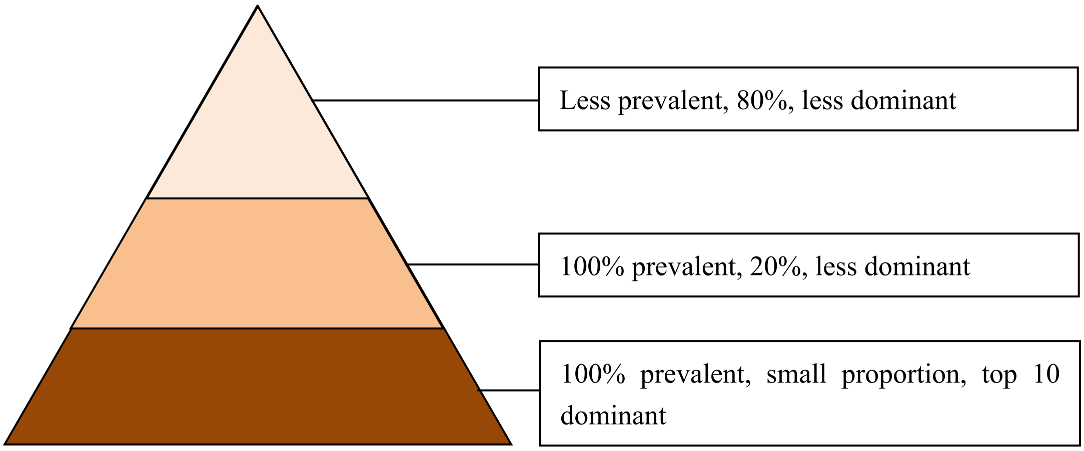

Figure S4. Gut microbiome prevalence. Bacterial prevalence and abundance varied across individuals and was host specific. In a specific segment intestine, about 80% of bacteria are less prevalent and 20% of bacteria are 100% prevalent across individuals. Very small portions of 100%-prevalent bacteria constitute the dominant microbiota. The abundance of dominant bacteria varied across individuals.
