# Supplementary material for: Structural and Functional Differences in Small Intestinal and Fecal Microbiota: 16S rRNA Gene Investigation in Rats
Source: Microorganisms. 2024 Aug 25;12(9):1764. doi: 10.3390/microorganisms12091764 (PMC11434385; doi:10.3390/microorganisms12091764)
Supplement: Supplementary file 1 [file microorganisms-12-01764-s001.zip › LetPub - Certificate 2023.pdf]

Certificate of English Language Editing

Manuscript Title:

Structural and Functional Differences in Small Intestinal and Fecal Microbiota: 16S rRNA Gene Investigation in Rats

Date of Revision:

April 11, 2023

Abstract:

Background: Intestinal microbiota changes are associated with various physiological and pathological processes and are considered a mechanism of disease. Most previous studies of intestinal microbiota were based on feces samples. With the development of 16S rRNA gene sequencing, however, several studies have shown that there is a difference between fecal and small intestinal microbiota, and that one cannot investigate gut microbiota with fecal samples. As the final form of food after digestion and absorption and as the final collection of intestinal flora, the extent to which the fecal microbiota can represent those of the whole intestine is still unknown. The differentiation of small-intestine microbiota from large-intestine microbiota might also hold great significance for disease occurrence.

Methods: To compare the differences in floral composition and functions between the two types of microbiota, ileal contents and feces were collected from Sprague Dawley (SD) rats fed in a conventional or specific-pathogen free (SPF) environment or from rats fed a high-fat diet (HFD), and the V3–V4 region of the 16S ribosomal ribonucleic acid (rRNA) gene in these rats was amplified and sequenced…

This document certifies that the manuscript listed above was copy edited for English language by LetPub, with regard to grammar, punctuation, spelling, and clarity. All of our language editors are native English speakers with long-term experience in editing scientific and technical manuscripts. We are committed to leveling the playing field for researchers whose native language is not English.

- Documents receiving this certification should be regarded as having undergone professional editorial revision for English language before submission. However, the authors may accept or reject LetPub's suggestions and changes at their own discretion and LetPub does not have editorial control over the submitted documents.
- The language quality of the submitted document is the sole responsibility of the submitting authors subject to those authors' adherence to LetPub's revisions and instruction. LetPub's provision of service does not constitute a guarantee or endorsement of the authors' work herein.
- Neither the research content nor the authors' intended meaning were altered in any way during the editing process.
- If you have any questions or concerns about this edited document, please contact us at support@letpub.com

LetPub Your Manuscript Editor

LetPub is an author service brand owned and operated by Accdon LLC. Headquartered in the Boston area, we are a full-spectrum author services company with a large team of US-based certified language and scientific editors, ISO 17001 accredited translators, and professional scientific illustrators and animators. We advocate ethical publication practices and are an official member of the Committee on Publication Ethics (COPE).

For more information about our company, services, and partnership programs, please visit www.letpub.com.

© 2023 Accdon, LLC. All Rights Reserved. Tel: 1-781-202-9968 Email: info@accdon.com Address: 400 Fifth Ave, Suite 530, Waltham, MA 02451, United States
