# Supplementary material for: Structural and Functional Differences in Small Intestinal and Fecal Microbiota: 16S rRNA Gene Investigation in Rats
Source: Microorganisms. 2024 Aug 25;12(9):1764. doi: 10.3390/microorganisms12091764 (PMC11434385; doi:10.3390/microorganisms12091764)
Supplement: Supplementary file 1 [file microorganisms-12-01764-s001.zip › Supplementary table 1.pdf]

Table S1. Fecal and ileal bacteria in chow fed rats under conventional environment (*n* = 6) (genus level)

	Taxonomy	Feces	Ileum
1	Unassigned; Unassigned; Unassigned; Unassigned; Unassigned; Unassigned;	0	3
2	k__Bacteria; p__Actinobacteria; c__Actinobacteria; o__Micrococcales; f__Microbacteriaceae; g__Candidatus_Aquiluna;	0	3
3	k__Bacteria; p__Acidobacteria; c__Aminicenantia; o__Aminicenantales; f__uncultured_bacterium_o_Aminicenantales; g__uncultured_bacterium_o_Aminicenantales;	0	4
4	k__Bacteria; p__Acidobacteria; c__Thermoanaerobaculia; o__Thermoanaerobaculales; f__Thermoanaerobaculaceae; g__Subgroup_10;	0	4
5	k__Bacteria; p__Actinobacteria; c__Actinobacteria; o__Micromonosporales; f__Micromonosporaceae; g__Luedemannella;	0	4
6	k__Bacteria; p__Firmicutes; c__Clostridia; o__Clostridiales; f__Ruminococcaceae; g__Ruminiclostridium_1;	0	4
7	k__Bacteria; p__Firmicutes; c__Bacilli; o__Bacillales; f__Staphylococcaceae; g__Aliicoccus;	0	5
8	k__Bacteria; p__Proteobacteria; c__Alphaproteobacteria; o__Reyranellales; f__Reyranellaceae; g__uncultured_bacterium_f_Reyranellaceae;	0	5
9	k__Bacteria; p__Proteobacteria; c__Gammaproteobacteria; o__Betaproteobacteriales; f__Burkholderiaceae; g__Comamonas;	0	5
10	k__Bacteria; p__Actinobacteria; c__Actinobacteria; o__Pseudonocardiales; f__Pseudonocardiaceae; g__Saccharopolyspora;	0	6

11	k__Bacteria; p__Firmicutes; c__Erysipelotrichia; o__Erysipelotrichales; f__Erysipelotrichaceae; g__Solobacterium;	0	6
12	k__Bacteria; p__Proteobacteria; c__Alphaproteobacteria; o__Rhizobiales; f__D05-2; g__uncultured_bacterium_f_D05-2;	0	6
13	k__Bacteria; p__Firmicutes; c__Clostridia; o__Clostridiales; f__Family_XI; g__W5053;	0	7
14	k__Bacteria; p__Proteobacteria; c__Alphaproteobacteria; o__Rhizobiales; f__Beijerinckiaceae; g__Methylocystis;	0	7
15	k__Bacteria; p__Proteobacteria; c__Deltaproteobacteria; o__Myxococcales; f__Haliangiaceae; g__Haliangium;	0	7
16	k__Bacteria; p__Planctomycetes; c__Planctomycetacia; o__Isosphaerales; f__Isosphaeraceae; g__uncultured_bacterium_f_Isosphaeraceae;	0	8
17	k__Bacteria; p__Proteobacteria; c__Gammaproteobacteria; o__Betaproteobacteriales; f__Burkholderiaceae; g__Variovorax;	0	8
18	k__Bacteria; p__Acidobacteria; c__Acidobacteriia; o__Solibacterales; f__Solibacteraceae_Subgroup_3; g__AKIW659;	0	9
19	k__Bacteria; p__Chloroflexi; c__Anaerolineae; o__Anaerolineales; f__Anaerolineaceae; g__Anaerolinea;	0	9
20	k__Bacteria; p__Proteobacteria; c__Gammaproteobacteria; o__Xanthomonadales; f__Rhodanobacteraceae; g__Rhodanobacter;	0	9
21	k__Bacteria; p__Bacteroidetes; c__Bacteroidia; o__Bacteroidales; f__F082; g__uncultured_bacterium_f_F082;	0	10
22	k__Bacteria; p__Proteobacteria; c__Alphaproteobacteria; o__Azospirillales; f__uncultured_bacterium_o_Azospirillales; g__uncultured_bacterium_o_Azospirillales;	0	10
23	k__Bacteria; p__Proteobacteria; c__Alphaproteobacteria; o__Rhizobiales; f__Rhizobiaceae; g__Pseudaminobacter;	0	10

24	k__Bacteria; p__Proteobacteria; c__Alphaproteobacteria; o__Rhodospirillales; f__Rhodopirillaceae; g__Defluviicoccus;	0	10
25	k__Bacteria; p__Verrucomicrobia; c__Verrucomicrobiae; o__Pedosphaerales; f__Pedosphaeraceae; g__ADurb.Bin063-1;	0	10
26	k__Bacteria; p__Bacteroidetes; c__Bacteroidia; o__Chitinophagales; f__Chitinophagaceae; g__Flavitalea;	0	11
27	k__Bacteria; p__Firmicutes; c__Negativicutes; o__Selenomonadales; f__Veillonellaceae; g__Selenomonas_3;	0	11
28	k__Bacteria; p__Proteobacteria; c__Alphaproteobacteria; o__Rhizobiales; f__Beijerinckiaceae; g__Roseiarcus;	0	11
29	k__Bacteria; p__Proteobacteria; c__Deltaproteobacteria; o__MBNT15; f__uncultured_bacterium_o_MBNT15; g__uncultured_bacterium_o_MBNT15;	0	11
30	k__Bacteria; p__Proteobacteria; c__Gammaproteobacteria; o__Betaproteobacteriales; f__Burkholderiaceae; g__Noviherbaspirillum;	0	11
31	k__Bacteria; p__Proteobacteria; c__Gammaproteobacteria; o__Betaproteobacteriales; f__Burkholderiaceae; g__Pelomonas;	0	12
32	k__Bacteria; p__Actinobacteria; c__Actinobacteria; o__Pseudonocardiales; f__Pseudonocardiaceae; g__uncultured_bacterium_f_Pseudonocardiaceae;	0	13
33	k__Bacteria; p__Proteobacteria; c__Deltaproteobacteria; o__Myxococcales; f__Archangiaceae; g__Anaeromyxobacter;	0	13
34	k__Bacteria; p__Actinobacteria; c__Actinobacteria; o__Micrococcales; f__Micrococcaceae; g__Kocuria;	0	14
35	k__Bacteria; p__Latescibacteria; c__uncultured_bacterium_p_Latescibacteria; o__uncultured_bacterium_p_Latescibacteria; f__uncultured_bacterium_p_Latescibacteria; g__uncultured_bacterium_p_Latescibacteria;	0	14

36	k__Bacteria; p__Proteobacteria; c__Alphaproteobacteria; o__Sphingomonadales; f__Sphingomonadaceae; g__Sphingopyxis;	0	14
37	k__Bacteria; p__Proteobacteria; c__Deltaproteobacteria; o__RCP2-54; f__uncultured_bacterium_o_RCP2-54; g__uncultured_bacterium_o_RCP2-54;	0	14
38	k__Bacteria; p__Bacteroidetes; c__Bacteroidia; o__Flavobacteriales; f__Weeksellaceae; g__uncultured_bacterium_f_Weeksellaceae;	0	15
39	k__Bacteria; p__Chloroflexi; c__P2-11E; o__uncultured_bacterium_c_P2-11E; f__uncultured_bacterium_c_P2-11E; g__uncultured_bacterium_c_P2-11E;	0	15
40	k__Bacteria; p__Cyanobacteria; c__Oxyphotobacteria; o__Synechococcales; f__Cyanobiaceae; g__Cyanobium_PCC-6307;	0	15
41	k__Bacteria; p__Proteobacteria; c__Alphaproteobacteria; o__Rhizobiales; f__Rhodomicrobiaceae; g__Rhodomicrobium;	0	15
42	k__Bacteria; p__Bacteroidetes; c__Bacteroidia; o__Bacteroidales; f__Muribaculaceae; g__CAG-873;	0	16
43	k__Bacteria; p__Firmicutes; c__Bacilli; o__Lactobacillales; f__Carnobacteriaceae; g__Lacticigenium;	0	16
44	k__Bacteria; p__Proteobacteria; c__Alphaproteobacteria; o__Sphingomonadales; f__Sphingomonadaceae; g__Altererythrobacter;	0	16
45	k__Bacteria; p__Proteobacteria; c__Deltaproteobacteria; o__Myxococcales; f__Phaselicystidaceae; g__Phaselicystis;	0	16
46	k__Bacteria; p__Proteobacteria; c__Gammaproteobacteria; o__Aeromonadales; f__Aeromonadaceae; g__Aeromonas;	0	16
47	k__Bacteria; p__Acidobacteria; c__Acidobacteriia; o__Solibacterales; f__Solibacteraceae_Subgroup_3; g__Paludibaculum;	0	17
48	k__Bacteria; p__Actinobacteria; c__Actinobacteria; o__Actinomycetales; f__Actinomycetaceae; g__Trueperella;	0	17

49	k__Bacteria; p__Actinobacteria; c__MB-A2-108; o__uncultured_bacterium_c_MB-A2-108; f__uncultured_bacterium_c_MB-A2-108; g__uncultured_bacterium_c_MB-A2-108;	0	17
50	k__Bacteria; p__Chloroflexi; c__Anaerolineae; o__SBR1031; f__A4b; g__uncultured_bacterium_f_A4b;	0	17
51	k__Bacteria; p__Cyanobacteria; c__Oxyphotobacteria; o__Chloroplast; f__Lolium_perenne; g__Lolium_perenne;	0	17
52	k__Bacteria; p__Gemmatimonadetes; c__Longimicrobia; o__Longimicrobiales; f__Longimicrobiaceae; g__uncultured_bacterium_f_Longimicrobiaceae;	0	17
53	k__Bacteria; p__Proteobacteria; c__Deltaproteobacteria; o__Myxococcales; f__P3OB-42; g__uncultured_bacterium_f_P3OB-42;	0	17
54	k__Bacteria; p__Proteobacteria; c__Gammaproteobacteria; o__Aeromonadales; f__Succinivibrionaceae; g__Succinivibrionaceae_UCG-001;	0	17
55	k__Bacteria; p__Proteobacteria; c__Gammaproteobacteria; o__Betaproteobacteriales; f__Burkholderiaceae; g__Burkholderia-Caballeronia-Paraburkholderia;	0	17
56	k__Bacteria; p__Acidobacteria; c__Blastocatellia_Subgroup_4; o__Blastocatellales; f__Blastocatellaceae; g__Aridibacter;	0	18
57	k__Bacteria; p__Actinobacteria; c__Actinobacteria; o__Micrococcales; f__Dermabacteraceae; g__Brachybacterium;	0	18
58	k__Bacteria; p__Actinobacteria; c__Actinobacteria; o__Micrococcales; f__Intrasporangiaceae; g__uncultured_bacterium_f_Intrasporangiaceae;	0	18
59	k__Bacteria; p__Cyanobacteria; c__Oxyphotobacteria; o__Nostocales; f__Nostocaceae; g__Nostoc_PCC-8976;	0	18
60	k__Bacteria; p__Proteobacteria; c__Gammaproteobacteria; o__Betaproteobacteriales; f__Burkholderiaceae; g__Alcaligenes;	0	18
61	k__Bacteria; p__Proteobacteria; c__Gammaproteobacteria; o__Betaproteobacteriales; f__Rhodocyclaceae; g__Candidatus_Accumulibacter;	0	18

62	k__Bacteria; p__Proteobacteria; c__Gammaproteobacteria; o__Oceanospirillales; f__Halomonadaceae; g__Halomonas;	0	18
63	k__Bacteria; p__Firmicutes; c__Clostridia; o__Clostridiales; f__Peptostreptococcaceae; g__Paraclostridium;	0	19
64	k__Bacteria; p__Proteobacteria; c__Alphaproteobacteria; o__Rhizobiales; f__Methyloligellaceae; g__uncultured_bacterium_f_Methyloligellaceae;	0	20
65	k__Bacteria; p__Actinobacteria; c__Actinobacteria; o__Corynebacteriales; f__Mycobacteriaceae; g__Mycobacterium;	0	21
66	k__Bacteria; p__Actinobacteria; c__Actinobacteria; o__Micrococcales; f__Microbacteriaceae; g__Glaciihabitans;	0	21
67	k__Bacteria; p__Epsilonbacteraeota; c__Campylobacteria; o__Campylobacterales; f__Campylobacteraceae; g__Campylobacter;	0	21
68	k__Bacteria; p__Firmicutes; c__Clostridia; o__Clostridiales; f__Family_XI; g__Anaerosalibacter;	0	21
69	k__Bacteria; p__Proteobacteria; c__Alphaproteobacteria; o__Caulobacterales; f__Hyphomonadaceae; g__SWB02;	0	21
70	k__Bacteria; p__Proteobacteria; c__Deltaproteobacteria; o__NB1-j; f__uncultured_bacterium_o_NB1-j; g__uncultured_bacterium_o_NB1-j;	0	21
71	k__Bacteria; p__Actinobacteria; c__Actinobacteria; o__Corynebacteriales; f__Dietziaceae; g__Dietzia;	0	22
72	k__Bacteria; p__Actinobacteria; c__Thermoleophilia; o__Gaiellales; f__Gaiellaceae; g__Gaiella;	0	22
73	k__Bacteria; p__Firmicutes; c__Bacilli; o__Lactobacillales; f__Lactobacillaceae; g__Pediococcus;	0	22
74	k__Bacteria; p__Proteobacteria; c__Alphaproteobacteria; o__Rhizobiales; f__Rhizobiaceae; g__Ensifer;	0	22

75	k__Bacteria; p__Nitrospirae; c__4-29-1; o__uncultured_bacterium_c_4-29-1; f__uncultured_bacterium_c_4-29-1; g__uncultured_bacterium_c_4-29-1;	0	23
76	k__Bacteria; p__Proteobacteria; c__Gammaproteobacteria; o__Xanthomonadales; f__Rhodanobacteraceae; g__Dokdonella;	0	23
77	k__Bacteria; p__Firmicutes; c__Negativicutes; o__Selenomonadales; f__Veillonellaceae; g__Megamonas;	0	24
78	k__Bacteria; p__Proteobacteria; c__Alphaproteobacteria; o__Rhizobiales; f__Hyphomicrobiaceae; g__Hyphomicrobium;	0	24
79	k__Bacteria; p__Firmicutes; c__Negativicutes; o__Selenomonadales; f__Acidaminococcaceae; g__Succiniclasticum;	0	26
80	k__Bacteria; p__Verrucomicrobia; c__Verrucomicrobiae; o__Chthoniobacterales; f__Xiphinematobacteraceae; g__Candidatus_Xiphinematobacter;	0	26
81	k__Bacteria; p__Actinobacteria; c__Acidimicrobiia; o__Microtrichales; f__uncultured_bacterium_o_Microtrichales; g__uncultured_bacterium_o_Microtrichales;	0	27
82	k__Bacteria; p__Firmicutes; c__Negativicutes; o__Selenomonadales; f__Acidaminococcaceae; g__Phascolarctobacterium;	0	27
83	k__Bacteria; p__Proteobacteria; c__Alphaproteobacteria; o__Caulobacterales; f__Hyphomonadaceae; g__Hirschia;	0	27
84	k__Bacteria; p__Actinobacteria; c__Actinobacteria; o__Propionibacteriales; f__Nocardioidaceae; g__Kribbella;	0	28
85	k__Bacteria; p__Proteobacteria; c__Alphaproteobacteria; o__Rhizobiales; f__Beijerinckiaceae; g__Bosea;	0	28
86	k__Bacteria; p__Proteobacteria; c__Alphaproteobacteria; o__Rhizobiales; f__Devosiaceae; g__Devosia;	0	28
87	k__Bacteria; p__Actinobacteria; c__Actinobacteria; o__Streptomycetales; f__Streptomycetaceae; g__Streptomyces;	0	29

88	k__Bacteria; p__Proteobacteria; c__Alphaproteobacteria; o__Rhizobiales; f__Hyphomicrobiaceae; g__Pedomicrobium;	0	29
89	k__Bacteria; p__Proteobacteria; c__Gammaproteobacteria; o__Betaproteobacteriales; f__Burkholderiaceae; g__Ramlibacter;	0	29
90	k__Bacteria; p__Proteobacteria; c__Gammaproteobacteria; o__Betaproteobacteriales; f__Nitrosomonadaceae; g__GOUTA6;	0	29
91	k__Bacteria; p__Actinobacteria; c__Acidimicrobiia; o__Actinomarinales; f__uncultured_bacterium_o_Actinomarinales; g__uncultured_bacterium_o_Actinomarinales;	0	30
92	k__Bacteria; p__Actinobacteria; c__Actinobacteria; o__Micrococcales; f__Brevibacteriaceae; g__Brevibacterium;	0	30
93	k__Bacteria; p__Proteobacteria; c__Gammaproteobacteria; o__Xanthomonadales; f__Xanthomonadaceae; g__Pseudoxanthomonas;	0	30
94	k__Bacteria; p__Bacteroidetes; c__Bacteroidia; o__Bacteroidales; f__Bacteroidetes_vadinHA17; g__uncultured_bacterium_f_Bacteroidetes_vadinHA17;	0	31
95	k__Bacteria; p__Bacteroidetes; c__Bacteroidia; o__Bacteroidales; f__p-251-o5; g__uncultured_bacterium_f_p-251-o5;	0	31
96	k__Bacteria; p__Planctomycetes; c__Planctomycetacia; o__Isosphaerales; f__Isosphaeraceae; g__Aquisphaera;	0	31
97	k__Bacteria; p__Proteobacteria; c__Alphaproteobacteria; o__Rhizobiales; f__Rhizobiaceae; g__Mesorhizobium;	0	31
98	k__Bacteria; p__Proteobacteria; c__Gammaproteobacteria; o__Betaproteobacteriales; f__Burkholderiaceae; g__Massilia;	0	31
99	k__Bacteria; p__Chloroflexi; c__TK10; o__uncultured_bacterium_c_TK10; f__uncultured_bacterium_c_TK10; g__uncultured_bacterium_c_TK10;	0	32
100	k__Bacteria; p__Proteobacteria; c__Alphaproteobacteria; o__Rhizobiales; f__Rhizobiales_Incertae_Sedis; g__Bauldia;	0	33

101	k__Bacteria; p__Proteobacteria; c__Alphaproteobacteria; o__Rhodobacterales; f__Rhodobacteraceae; g__Rhodobacter;	0	33
102	k__Bacteria; p__Acidobacteria; c__Acidobacteriia; o__Acidobacteriales; f__Acidobacteriaceae_Subgroup_1; g__Occallatibacter;	0	34
103	k__Bacteria; p__Actinobacteria; c__Actinobacteria; o__Micrococcales; f__Microbacteriaceae; g__Leucobacter;	0	34
104	k__Bacteria; p__Firmicutes; c__Bacilli; o__Bacillales; f__Planococcaceae; g__Lysinibacillus;	0	34
105	k__Bacteria; p__Gemmatimonadetes; c__Gemmatimonadetes; o__Gemmatimonadales; f__Gemmatimonadaceae; g__Gemmatimonas;	0	34
106	k__Bacteria; p__Nitrospirae; c__Thermodesulfovibrionia; o__uncultured_bacterium_c_Thermodesulfovibrionia; f__uncultured_bacterium_c_Thermodesulfovibrionia; g__uncultured_bacterium_c_Thermodesulfovibrionia;	0	34
107	k__Bacteria; p__Proteobacteria; c__Gammaproteobacteria; o__Enterobacteriales; f__Enterobacteriaceae; g__Plesiomonas;	0	34
108	k__Bacteria; p__Proteobacteria; c__Alphaproteobacteria; o__Rhodobacterales; f__Rhodobacteraceae; g__Paracoccus;	0	36
109	k__Bacteria; p__Proteobacteria; c__Gammaproteobacteria; o__Betaproteobacteriales; f__A21b; g__uncultured_bacterium_f_A21b;	0	36
110	k__Bacteria; p__Proteobacteria; c__Alphaproteobacteria; o__Rhizobiales; f__A0839; g__uncultured_bacterium_f_A0839;	0	37
111	k__Bacteria; p__Proteobacteria; c__Alphaproteobacteria; o__Rhizobiales; f__Xanthobacteraceae; g__Pseudolabrys;	0	37
112	k__Bacteria; p__Bacteroidetes; c__Bacteroidia; o__Sphingobacteriales; f__Sphingobacteriaceae; g__Pedobacter;	0	38

113	k__Bacteria; p__Planctomycetes; c__Phycisphaerae; o__Phycisphaerales; f__Phycisphaeraceae; g__AKYG587;	0	38
114	k__Bacteria; p__Proteobacteria; c__Gammaproteobacteria; o__Betaproteobacteriales; f__Burkholderiaceae; g__uncultured_bacterium_f_Burkholderiaceae;	0	39
115	k__Bacteria; p__Proteobacteria; c__Gammaproteobacteria; o__CCD24; f__uncultured_bacterium_o_CCD24; g__uncultured_bacterium_o_CCD24;	0	39
116	k__Bacteria; p__Actinobacteria; c__Acidimicrobiia; o__Microtrichales; f__Iamiaceae; g__Iamia;	0	40
117	k__Bacteria; p__Proteobacteria; c__Alphaproteobacteria; o__Caulobacterales; f__Caulobacteraceae; g__Caulobacter;	0	40
118	k__Bacteria; p__Proteobacteria; c__Gammaproteobacteria; o__Betaproteobacteriales; f__Nitrosomonadaceae; g__IS-44;	0	40
119	k__Bacteria; p__Fusobacteria; c__Fusobacteriia; o__Fusobacteriales; f__Fusobacteriaceae; g__Fusobacterium;	0	43
120	k__Bacteria; p__Chloroflexi; c__AD3; o__uncultured_bacterium_c_AD3; f__uncultured_bacterium_c_AD3; g__uncultured_bacterium_c_AD3;	0	44
121	k__Bacteria; p__Proteobacteria; c__Alphaproteobacteria; o__Caulobacterales; f__Caulobacteraceae; g__Brevundimonas;	0	44
122	k__Bacteria; p__Proteobacteria; c__Gammaproteobacteria; o__Betaproteobacteriales; f__Hydrogenophilaceae; g__uncultured_bacterium_f_Hydrogenophilaceae;	0	44
123	k__Bacteria; p__Chloroflexi; c__Gitt-GS-136; o__uncultured_bacterium_c_Gitt-GS-136; f__uncultured_bacterium_c_Gitt-GS-136; g__uncultured_bacterium_c_Gitt-GS-136;	0	45
124	k__Bacteria; p__Proteobacteria; c__Alphaproteobacteria; o__uncultured_bacterium_c_Alphaproteobacteria; f__uncultured_bacterium_c_Alphaproteobacteria; g__uncultured_bacterium_c_Alphaproteobacteria;	0	45

125	k__Bacteria; p__Acidobacteria; c__Acidobacteriia; o__Subgroup_2; f__uncultured_bacterium_o_Subgroup_2; g__uncultured_bacterium_o_Subgroup_2;	0	47
126	k__Bacteria; p__Proteobacteria; c__Gammaproteobacteria; o__Gammaproteobacteria_Incertae_Sedis; Unclassified; g__Acidibacter;	0	47
127	k__Bacteria; p__Proteobacteria; c__Alphaproteobacteria; o__Rhizobiales; f__Rhizobiaceae; g__Allorhizobium-Neorhizobium-Pararhizobium-Rhizobium;	0	48
128	k__Bacteria; p__Acidobacteria; c__Acidobacteriia; o__Acidobacteriales; f__Koribacteraceae; g__Candidatus_Koribacter;	0	49
129	k__Bacteria; p__Bacteroidetes; c__Bacteroidia; o__Chitinophagales; f__Chitinophagaceae; g__uncultured_bacterium_f_Chitinophagaceae;	0	49
130	k__Bacteria; p__Actinobacteria; c__Actinobacteria; o__Frankiales; f__Acidothermaceae; g__Acidothermus;	0	50
131	k__Bacteria; p__Bacteroidetes; c__Bacteroidia; o__Bacteroidales; f__Rikenellaceae; g__hoa5-07d05_gut_group;	0	50
132	k__Bacteria; p__Firmicutes; c__Negativicutes; o__Selenomonadales; f__Veillonellaceae; g__Megasphaera;	0	51
133	k__Bacteria; p__Acidobacteria; c__Blastocatellia_Subgroup_4; o__11-24; f__uncultured_bacterium_o_11-24; g__uncultured_bacterium_o_11-24;	0	52
134	k__Bacteria; p__Bacteroidetes; c__Bacteroidia; o__Chitinophagales; f__Chitinophagaceae; g__Terrimonas;	0	53
135	k__Bacteria; p__Firmicutes; c__Clostridia; o__Clostridiales; f__Clostridiaceae_1; g__Clostridium_sensu_stricto_13;	0	54
136	k__Bacteria; p__Proteobacteria; c__Alphaproteobacteria; o__Rhizobiales; f__uncultured_bacterium_o_Rhizobiales; g__uncultured_bacterium_o_Rhizobiales;	0	54
137	k__Bacteria; p__Proteobacteria; c__Gammaproteobacteria; o__Betaproteobacteriales; f__Burkholderiaceae; g__Rhodoferax;	0	55

138	k__Bacteria; p__Acidobacteria; c__Subgroup_17; o__uncultured_bacterium_c_Subgroup_17; f__uncultured_bacterium_c_Subgroup_17; g__uncultured_bacterium_c_Subgroup_17;	0	57
139	k__Bacteria; p__Actinobacteria; c__Acidimicrobiia; o__uncultured_bacterium_c_Acidimicrobiia; f__uncultured_bacterium_c_Acidimicrobiia; g__uncultured_bacterium_c_Acidimicrobiia;	0	57
140	k__Bacteria; p__Actinobacteria; c__Thermoleophilia; o__Gaiellales; f__uncultured_bacterium_o_Gaiellales; g__uncultured_bacterium_o_Gaiellales;	0	57
141	k__Bacteria; p__Proteobacteria; c__Alphaproteobacteria; o__Rhizobiales; f__Rhizobiales_Incertae_Sedis; g__uncultured_bacterium_f_Rhizobiales_Incertae_Sedis;	0	57
142	k__Bacteria; p__Bacteroidetes; c__Bacteroidia; o__Bacteroidales; f__p-2534-18B5_gut_group; g__uncultured_bacterium_f_p-2534-18B5_gut_group;	0	58
143	k__Bacteria; p__Firmicutes; c__Bacilli; o__Lactobacillales; f__Enterococcaceae; g__Enterococcus;	0	58
144	k__Bacteria; p__Proteobacteria; c__Alphaproteobacteria; o__Sphingomonadales; f__Sphingomonadaceae; g__Sphingorhabdus;	0	58
145	k__Bacteria; p__Bacteroidetes; c__Bacteroidia; o__Chitinophagales; f__Chitinophagaceae; g__Ferruginibacter;	0	59
146	k__Bacteria; p__Bacteroidetes; c__Bacteroidia; o__Chitinophagales; f__Saprospiraceae; g__uncultured_bacterium_f_Saprospiraceae;	0	61
147	k__Bacteria; p__Bacteroidetes; c__Bacteroidia; o__Cytophagales; f__Microscillaceae; g__uncultured_bacterium_f_Microscillaceae;	0	63
148	k__Bacteria; p__Actinobacteria; c__Acidimicrobiia; o__IMCC26256; f__uncultured_bacterium_o_IMCC26256; g__uncultured_bacterium_o_IMCC26256;	0	66
149	k__Bacteria; p__Nitrospirae; c__Nitrospira; o__Nitrospirales; f__Nitrospiraceae; g__Nitrospira;	0	66
150	k__Bacteria; p__Proteobacteria; c__Alphaproteobacteria; o__Rhizobiales; f__Rhizobiales_Incertae_Sedis; g__Nordella;	0	69

151	k__Bacteria; p__Proteobacteria; c__Alphaproteobacteria; o__Sphingomonadales; f__Sphingomonadaceae; g__Novosphingobium;	0	69
152	k__Bacteria; p__Chloroflexi; c__Anaerolineae; o__Anaerolineales; f__Anaerolineaceae; g__uncultured_bacterium_f_Anaerolineaceae;	0	70
153	k__Bacteria; p__Proteobacteria; c__Alphaproteobacteria; o__Micropepsales; f__Micropepsaceae; g__uncultured_bacterium_f_Micropepsaceae;	0	70
154	k__Bacteria; p__Proteobacteria; c__Alphaproteobacteria; o__Dongiales; f__Dongiaceae; g__Dongia;	0	75
155	k__Bacteria; p__Proteobacteria; c__Alphaproteobacteria; o__Rhizobiales; f__KF-JG30-B3; g__uncultured_bacterium_f_KF-JG30-B3;	0	75
156	k__Bacteria; p__Actinobacteria; c__Acidimicrobiia; o__Microtrichales; f__Ilumatobacteraceae; g__uncultured_bacterium_f_Ilumatobacteraceae;	0	78
157	k__Bacteria; p__Proteobacteria; c__Alphaproteobacteria; o__Rhizobiales; f__Beijerinckiaceae; g__Methylobacterium;	0	78
158	k__Bacteria; p__Proteobacteria; c__Gammaproteobacteria; o__PLTA13; f__uncultured_bacterium_o_PLTA13; g__uncultured_bacterium_o_PLTA13;	0	79
159	k__Bacteria; p__Proteobacteria; c__Deltaproteobacteria; o__Myxococcales; f__bacteriap25; g__uncultured_bacterium_f_bacteriap25;	0	81
160	k__Bacteria; p__Firmicutes; c__Bacilli; o__Bacillales; f__Planococcaceae; g__Sporosarcina;	0	82
161	k__Bacteria; p__Proteobacteria; c__Gammaproteobacteria; o__Betaproteobacteriales; f__Nitrosomonadaceae; g__MND1;	0	82
162	k__Bacteria; p__Acidobacteria; c__Blastocatellia_Subgroup_4; o__Blastocatellales; f__Blastocatellaceae; g__uncultured_bacterium_f_Blastocatellaceae;	0	92
163	k__Bacteria; p__Proteobacteria; c__Alphaproteobacteria; o__Rhizobiales; f__Xanthobacteraceae; g__Rhodoplanes;	0	93

164	k__Bacteria; p__Proteobacteria; c__Gammaproteobacteria; o__Betaproteobacteriales; f__Burkholderiaceae; g__Rhizobacter;	0	93
165	k__Bacteria; p__Acidobacteria; c__Blastocatellia_Subgroup_4; o__Blastocatellales; f__Blastocatellaceae; g__JGI_0001001-H03;	0	96
166	k__Bacteria; p__Firmicutes; c__Bacilli; o__Lactobacillales; f__Leuconostocaceae; g__Weissella;	0	97
167	k__Bacteria; p__Proteobacteria; c__Gammaproteobacteria; o__Xanthomonadales; f__Xanthomonadaceae; g__Lysobacter;	0	97
168	k__Bacteria; p__Proteobacteria; c__Alphaproteobacteria; o__Caulobacterales; f__Caulobacteraceae; g__Phenylobacterium;	0	102
169	k__Bacteria; p__Proteobacteria; c__Gammaproteobacteria; o__Xanthomonadales; f__Xanthomonadaceae; g__Thermomonas;	0	111
170	k__Bacteria; p__Proteobacteria; c__Alphaproteobacteria; o__Reyranellales; f__Reyranellaceae; g__Reyranella;	0	113
171	k__Bacteria; p__Proteobacteria; c__Gammaproteobacteria; o__Pseudomonadales; f__Pseudomonadaceae; g__Pseudomonas;	0	115
172	k__Bacteria; p__Proteobacteria; c__Gammaproteobacteria; o__Betaproteobacteriales; f__SC-I-84; g__uncultured_bacterium_f_SC-I-84;	0	120
173	k__Bacteria; p__Proteobacteria; c__Gammaproteobacteria; o__Betaproteobacteriales; f__Nitrosomonadaceae; g__Ellin6067;	0	122
174	k__Bacteria; p__Acidobacteria; c__Acidobacteriia; o__Solibacterales; f__Solibacteraceae_Subgroup_3; g__Bryobacter;	0	124
175	k__Bacteria; p__Acidobacteria; c__Holophagae; o__Subgroup_7; f__uncultured_bacterium_o_Subgroup_7; g__uncultured_bacterium_o_Subgroup_7;	0	134
176	k__Bacteria; p__Firmicutes; c__Clostridia; o__Clostridiales; f__Ruminococcaceae; g__Faecalibacterium;	0	136

177	k__Bacteria; p__Proteobacteria; c__Gammaproteobacteria; o__Betaproteobacteriales; f__TRA3-20; g__uncultured_bacterium_f_TRA3-20;	0	139
178	k__Bacteria; p__Firmicutes; c__Clostridia; o__Clostridiales; f__Clostridiaceae_1; g__Sarcina;	0	146
179	k__Bacteria; p__Bacteroidetes; c__Bacteroidia; o__Flavobacteriales; f__Flavobacteriaceae; g__Flavobacterium;	0	162
180	k__Bacteria; p__Fusobacteria; c__Fusobacteriia; o__Fusobacteriales; f__Fusobacteriaceae; g__Cetobacterium;	0	164
181	k__Bacteria; p__Proteobacteria; c__Alphaproteobacteria; o__Rhizobiales; f__Rhizobiaceae; g__Ochrobactrum;	0	193
182	k__Bacteria; p__Rokubacteria; c__NC10; o__Rokubacteriales; f__uncultured_bacterium_o_Rokubacteriales; g__uncultured_bacterium_o_Rokubacteriales;	0	198
183	k__Bacteria; p__Acidobacteria; c__Acidobacteriia; o__Acidobacteriales; f__uncultured_bacterium_o_Acidobacteriales; g__uncultured_bacterium_o_Acidobacteriales;	0	217
184	k__Bacteria; p__Proteobacteria; c__Gammaproteobacteria; o__Pseudomonadales; f__Moraxellaceae; g__Acinetobacter;	0	226
185	k__Bacteria; p__Verrucomicrobia; c__Verrucomicrobiae; o__Chthoniobacterales; f__Chthoniobacteraceae; g__Candidatus_Udaeobacter;	0	226
186	k__Bacteria; p__Proteobacteria; c__Gammaproteobacteria; o__Pasteurellales; f__Pasteurellaceae; g__Muribacter;	0	233
187	k__Bacteria; p__Proteobacteria; c__Alphaproteobacteria; o__Sphingomonadales; f__Sphingomonadaceae; g__uncultured_bacterium_f_Sphingomonadaceae;	0	236
188	k__Bacteria; p__Chloroflexi; c__KD4-96; o__uncultured_bacterium_c_KD4-96; f__uncultured_bacterium_c_KD4-96; g__uncultured_bacterium_c_KD4-96;	0	300
189	k__Bacteria; p__Proteobacteria; c__Alphaproteobacteria; o__Rhizobiales; f__Xanthobacteraceae; g__Bradyrhizobium;	0	307

190	k__Bacteria; p__Acidobacteria; c__Blastocatellia_Subgroup_4; o__Pyrinomonadales; f__Pyrinomonadaceae; g__RB41;	0	684
191	k__Bacteria; p__Acidobacteria; c__Subgroup_6; o__uncultured_bacterium_c_Subgroup_6; f__uncultured_bacterium_c_Subgroup_6; g__uncultured_bacterium_c_Subgroup_6;	0	909
192	k__Bacteria; p__Actinobacteria; c__Actinobacteria; o__Corynebacteriales; f__Nocardiaceae; g__Rhodococcus;	0	951
193	k__Bacteria; p__Proteobacteria; c__Alphaproteobacteria; o__Acetobacterales; f__Acetobacteraceae; g__Acetobacter;	0	1798
194	k__Bacteria; p__Firmicutes; c__Bacilli; o__Lactobacillales; f__Streptococcaceae; g__Lactococcus;	1	11
195	k__Bacteria; p__Firmicutes; c__Erysipelotrichia; o__Erysipelotrichales; f__Erysipelotrichaceae; g__Holdemanella;	1	18
196	k__Bacteria; p__Proteobacteria; c__Alphaproteobacteria; o__Sphingomonadales; f__Sphingomonadaceae; g__Plot4-2H12;	1	21
197	k__Bacteria; p__Cyanobacteria; c__Oxyphotobacteria; o__Chloroplast; f__uncultured_bacterium; g__uncultured_bacterium;	1	41
198	k__Bacteria; p__Firmicutes; c__Clostridia; o__Clostridiales; f__Lachnospiraceae; g__Lachnospiraceae_AC2044_group;	1	43
199	k__Bacteria; p__Actinobacteria; c__Actinobacteria; o__Micrococcales; f__Micrococcaceae; g__Arthrobacter;	1	95
200	k__Bacteria; p__Actinobacteria; c__Actinobacteria; o__Propionibacteriales; f__Propionibacteriaceae; g__Cutibacterium;	1	99
201	k__Bacteria; p__Firmicutes; c__Bacilli; o__Bacillales; f__Staphylococcaceae; g__Staphylococcus;	1	136
202	k__Bacteria; p__Actinobacteria; c__Actinobacteria; o__Corynebacteriales; f__Corynebacteriaceae; g__Corynebacterium_1;	1	149

203	k__Bacteria; p__Gemmatimonadetes; c__Gemmatimonadetes; o__Gemmatimonadales; f__Gemmatimonadaceae; g__uncultured_bacterium_f_Gemmatimonadaceae;	1	149
204	k__Bacteria; p__Actinobacteria; c__Acidimicrobiia; o__Microtrichales; f__Ilumatobacteraceae; g__CL500-29_marine_group;	1	239
205	k__Bacteria; p__Acidobacteria; c__Acidobacteriia; o__Solibacterales; f__Solibacteraceae_Subgroup_3; g__Candidatus_Solibacter;	1	246
206	k__Bacteria; p__Firmicutes; c__Bacilli; o__Lactobacillales; f__Carnobacteriaceae; g__Carnobacterium;	1	306
207	k__Bacteria; p__Actinobacteria; c__Actinobacteria; o__Bifidobacteriales; f__Bifidobacteriaceae; g__Bifidobacterium;	1	307
208	k__Bacteria; p__Proteobacteria; c__Gammaproteobacteria; o__Xanthomonadales; f__Xanthomonadaceae; g__Arenimonas;	1	326
209	k__Bacteria; p__Proteobacteria; c__Alphaproteobacteria; o__Rhizobiales; f__Xanthobacteraceae; g__uncultured_bacterium_f_Xanthobacteraceae;	1	333
210	k__Bacteria; p__Firmicutes; c__Clostridia; o__Clostridiales; f__Lachnospiraceae; g__Lachnospiraceae_XPB1014_group;	1	381
211	k__Bacteria; p__Firmicutes; c__Clostridia; o__Clostridiales; f__Peptostreptococcaceae; g__Terrisporobacter;	1	636
212	k__Bacteria; p__Proteobacteria; c__Alphaproteobacteria; o__Sphingomonadales; f__Sphingomonadaceae; g__Sphingomonas;	1	872
213	k__Bacteria; p__Firmicutes; c__Bacilli; o__Bacillales; f__Staphylococcaceae; g__Jeotgalicoccus;	2	39
214	k__Bacteria; p__Proteobacteria; c__Gammaproteobacteria; o__Steroidobacterales; f__Steroidobacteraceae; g__uncultured_bacterium_f_Steroidobacteraceae;	2	52
215	k__Bacteria; p__Firmicutes; c__Clostridia; o__Clostridiales; f__Clostridiales_vadinBB60_group; g__uncultured_bacterium_f_Clostridiales_vadinBB60_group;	3	19

216	k__Bacteria; p__Firmicutes; c__Bacilli; o__Lactobacillales; f__Aerococcaceae; g__Globicatella;	3	136
217	k__Bacteria; p__Cyanobacteria; c__Oxyphotobacteria; o__Chloroplast; f__Nicotiana_otophora; g__Nicotiana_otophora;	3	265
218	k__Bacteria; p__Firmicutes; c__Clostridia; o__Clostridiales; f__Ruminococcaceae; g__Oscillospira;	4	17
219	k__Bacteria; p__Firmicutes; c__Bacilli; o__Bacillales; f__Bacillaceae; g__Bacillus;	4	464
220	k__Bacteria; p__Firmicutes; c__Erysipelotrichia; o__Erysipelotrichales; f__Erysipelotrichaceae; g__Faecalibaculum;	4	520
221	k__Bacteria; p__Firmicutes; c__Erysipelotrichia; o__Erysipelotrichales; f__Erysipelotrichaceae; g__Allobaculum;	5	34
222	k__Bacteria; p__Firmicutes; c__Bacilli; o__Lactobacillales; f__Carnobacteriaceae; g__uncultured_bacterium_f_Carnobacteriaceae;	5	50
223	k__Bacteria; p__Actinobacteria; c__Actinobacteria; o__Corynebacteriales; f__Corynebacteriaceae; g__Corynebacterium;	5	226
224	k__Bacteria; p__Proteobacteria; c__Gammaproteobacteria; o__Pasteurellales; f__Pasteurellaceae; g__Rodentibacter;	6	1504
225	k__Bacteria; p__Firmicutes; c__Bacilli; o__Bacillales; f__Family_XI; g__Gemella;	8	383
226	k__Bacteria; p__Proteobacteria; c__Gammaproteobacteria; o__Enterobacteriales; f__Enterobacteriaceae; g__Enterobacter;	11	105
227	k__Bacteria; p__Firmicutes; c__Negativicutes; o__Selenomonadales; f__Veillonellaceae; g__Veillonella;	14	1018
228	k__Bacteria; p__Firmicutes; c__Clostridia; o__Clostridiales; f__Lachnospiraceae; g__A2;	15	3

229	k__Bacteria; p__Firmicutes; c__Erysipelotrichia; o__Erysipelotrichales; f__Erysipelotrichaceae; g__Erysipelotrichaceae_UCG-003;	15	4
230	k__Bacteria; p__Firmicutes; c__Clostridia; o__Clostridiales; f__Ruminococcaceae; g__Ruminococcaceae_UCG-002;	15	394
231	k__Bacteria; p__Firmicutes; c__Erysipelotrichia; o__Erysipelotrichales; f__Erysipelotrichaceae; g__Candidatus_Stoquefichus;	16	0
232	k__Bacteria; p__Bacteroidetes; c__Bacteroidia; o__Bacteroidales; f__Rs-E47_termite_group; g__uncultured_bacterium_f_Rs-E47_termite_group;	19	1
233	k__Bacteria; p__Firmicutes; c__Clostridia; o__Clostridiales; f__Ruminococcaceae; g__Negativibacillus;	22	26
234	k__Bacteria; p__Firmicutes; c__Clostridia; o__Clostridiales; f__Ruminococcaceae; g__Ruminococcaceae_UCG-010;	22	33
235	k__Bacteria; p__Firmicutes; c__Bacilli; o__Lactobacillales; f__Lactobacillaceae; g__Lactobacillus;	23	4598
236	k__Bacteria; p__Firmicutes; c__Clostridia; o__Clostridiales; f__Clostridiaceae_1; g__Candidatus_Arthromitus;	24	1466
237	k__Bacteria; p__Firmicutes; c__Clostridia; o__Clostridiales; f__Lachnospiraceae; g__ASF356;	28	14
238	k__Bacteria; p__Actinobacteria; c__Coriobacteriia; o__Coriobacteriales; f__Coriobacteriaceae; g__Collinsella;	28	18
239	k__Bacteria; p__Proteobacteria; c__Gammaproteobacteria; o__Enterobacteriales; f__Enterobacteriaceae; g__Escherichia-Shigella;	29	3251
240	k__Bacteria; p__Firmicutes; c__Clostridia; o__Clostridiales; f__Ruminococcaceae; g__Anaerotruncus;	32	20
241	k__Bacteria; p__Actinobacteria; c__Coriobacteriia; o__Coriobacteriales; f__Atopobiaceae; g__uncultured_bacterium_f_Atopobiaceae;	32	22

242	k__Bacteria; p__Firmicutes; c__Clostridia; o__Clostridiales; f__Lachnospiraceae; g__Eisenbergiella;	34	0
243	k__Bacteria; p__Epsilonbacteraeota; c__Campylobacteria; o__Campylobacterales; f__Helicobacteraceae; g__Helicobacter;	36	1305
244	k__Bacteria; p__Firmicutes; c__Clostridia; o__Clostridiales; f__Ruminococcaceae; g__Candidatus_Soleaferrea;	38	2
245	k__Bacteria; p__Bacteroidetes; c__Bacteroidia; o__Bacteroidales; f__Prevotellaceae; g__Prevotellaceae_NK3B31_group;	41	175
246	k__Bacteria; p__Actinobacteria; c__Coriobacteriia; o__Coriobacteriales; f__Atopobiaceae; g__Coriobacteriaceae_UCG-002;	44	30
247	k__Bacteria; p__Firmicutes; c__Clostridia; o__Clostridiales; f__Peptococcaceae; g__uncultured_bacterium_f_Peptococcaceae;	44	39
248	k__Bacteria; p__Proteobacteria; c__Deltaproteobacteria; o__Desulfovibrionales; f__Desulfovibrionaceae; g__Bilophila;	47	39
249	k__Bacteria; p__Actinobacteria; c__Actinobacteria; o__Actinomycetales; f__Actinomycetaceae; g__Actinomyces;	53	237
250	k__Bacteria; p__Firmicutes; c__Clostridia; o__Clostridiales; f__Lachnospiraceae; g__Coprococcus_3;	54	47
251	k__Bacteria; p__Proteobacteria; c__Alphaproteobacteria; o__Rhodospirillales; f__uncultured_bacterium_o_Rhodospirillales; g__uncultured_bacterium_o_Rhodospirillales;	56	7
252	k__Bacteria; p__Firmicutes; c__Clostridia; o__Clostridiales; f__Peptococcaceae; g__Peptococcus;	56	64
253	k__Bacteria; p__Actinobacteria; c__Coriobacteriia; o__Coriobacteriales; f__Eggerthellaceae; g__Gordonibacter;	64	4
254	k__Bacteria; p__Firmicutes; c__Clostridia; o__Clostridiales; f__Ruminococcaceae; g__Papillibacter;	65	40

255	k__Bacteria; p__Firmicutes; c__Clostridia; o__Clostridiales; f__Family_XIII; g__[Eubacterium]_brachy_group;	66	24
256	k__Bacteria; p__Firmicutes; c__Clostridia; o__Clostridiales; f__Lachnospiraceae; g__[Eubacterium]_ventriosum_group;	72	32
257	k__Bacteria; p__Actinobacteria; c__Coriobacteriia; o__Coriobacteriales; f__Eggerthellaceae; g__Adlercreutzia;	74	9
258	k__Bacteria; p__Firmicutes; c__Clostridia; o__Clostridiales; f__Ruminococcaceae; g__Anaerofilum;	74	12
259	k__Bacteria; p__Firmicutes; c__Clostridia; o__Clostridiales; f__Christensenellaceae; g__uncultured_bacterium_f_Christensenellaceae;	75	16
260	k__Bacteria; p__Firmicutes; c__Clostridia; o__Clostridiales; f__Ruminococcaceae; g__Butyricicoccus;	79	31
261	k__Bacteria; p__Bacteroidetes; c__Bacteroidia; o__Bacteroidales; f__Prevotellaceae; g__Prevotella_1;	81	43
262	k__Bacteria; p__Firmicutes; c__Clostridia; o__Clostridiales; f__Defluviitaleaceae; g__Defluviitaleaceae_UCG-011;	90	0
263	k__Bacteria; p__Firmicutes; c__Clostridia; o__Clostridiales; f__Lachnospiraceae; g__Lachnospiraceae_NK4B4_group;	99	11
264	k__Bacteria; p__Proteobacteria; c__Gammaproteobacteria; o__Betaproteobacteriales; f__Burkholderiaceae; g__Parasutterella;	108	198
265	k__Bacteria; p__Firmicutes; c__Clostridia; o__Clostridiales; f__Family_XIII; g__[Eubacterium]_nodatum_group;	117	18
266	k__Bacteria; p__Firmicutes; c__Bacilli; o__Lactobacillales; f__Streptococcaceae; g__Streptococcus;	136	4771
267	k__Bacteria; p__Actinobacteria; c__Coriobacteriia; o__Coriobacteriales; f__uncultured_bacterium_o_Coriobacteriales; g__uncultured_bacterium_o_Coriobacteriales;	142	41

268	k__Bacteria; p__Actinobacteria; c__Coriobacteriia; o__Coriobacteriales; f__Eggerthellaceae; g__Parvibacter;	147	21
269	k__Bacteria; p__Firmicutes; c__Clostridia; o__Clostridiales; f__Lachnospiraceae; g__[Eubacterium]_oxidoreducens_group;	148	0
270	k__Bacteria; p__Firmicutes; c__Clostridia; o__Clostridiales; f__Lachnospiraceae; g__Lachnospiraceae_NC2004_group;	155	19
271	k__Bacteria; p__Firmicutes; c__Clostridia; o__Clostridiales; f__Lachnospiraceae; g__Dorea;	155	37
272	k__Bacteria; p__Bacteroidetes; c__Bacteroidia; o__Bacteroidales; f__Marinifilaceae; g__Odoribacter;	164	6
273	k__Bacteria; p__Firmicutes; c__Clostridia; o__Clostridiales; f__Lachnospiraceae; g__Marvinbryantia;	170	57
274	k__Bacteria; p__Bacteroidetes; c__Bacteroidia; o__Bacteroidales; f__Prevotellaceae; g__Prevotella_9;	170	331
275	k__Bacteria; p__Bacteroidetes; c__Bacteroidia; o__Bacteroidales; f__Prevotellaceae; g__Alloprevotella;	183	199
276	k__Bacteria; p__Firmicutes; c__Clostridia; o__Clostridiales; f__Ruminococcaceae; g__UBA1819;	203	32
277	k__Bacteria; p__Firmicutes; c__Clostridia; o__Clostridiales; f__Family_XIII; g__Family_XIII_UCG-001;	206	60
278	k__Bacteria; p__Firmicutes; c__Clostridia; o__Clostridiales; f__Lachnospiraceae; g__Lachnospiraceae_UCG-008;	216	12
279	k__Bacteria; p__Actinobacteria; c__Coriobacteriia; o__Coriobacteriales; f__Eggerthellaceae; g__Enterorhabdus;	220	52
280	k__Bacteria; p__Firmicutes; c__Clostridia; o__Clostridiales; f__Lachnospiraceae; g__Tyzzerella;	222	27

281	k__Bacteria; p__Firmicutes; c__Clostridia; o__Clostridiales; f__Lachnospiraceae; g__Blautia;	281	99
282	k__Bacteria; p__Firmicutes; c__Clostridia; o__Clostridiales; f__Ruminococcaceae; g__Subdoligranulum;	305	53
283	k__Bacteria; p__Actinobacteria; c__Coriobacteriia; o__Coriobacteriales; f__Eggerthellaceae; g__DNF00809;	312	33
284	k__Bacteria; p__Bacteroidetes; c__Bacteroidia; o__Bacteroidales; f__Tannerellaceae; g__Parabacteroides;	335	62
285	k__Bacteria; p__Firmicutes; c__Erysipelotrichia; o__Erysipelotrichales; f__Erysipelotrichaceae; g__uncultured_bacterium_f_Erysipelotrichaceae;	353	156
286	k__Bacteria; p__Firmicutes; c__Clostridia; o__Clostridiales; f__Family_XIII; g__Anaerovorax;	404	106
287	k__Bacteria; p__Firmicutes; c__Clostridia; o__Clostridiales; f__Ruminococcaceae; g__Ruminococcaceae_UCG-009;	409	81
288	k__Bacteria; p__Bacteroidetes; c__Bacteroidia; o__Bacteroidales; f__Rikenellaceae; g__Alistipes;	429	1601
289	k__Bacteria; p__Firmicutes; c__Clostridia; o__Clostridiales; f__Ruminococcaceae; g__Ruminiclostridium;	512	77
290	k__Bacteria; p__Cyanobacteria; c__Melainabacteria; o__Gastranaerophilales; f__uncultured_bacterium_o_Gastranaerophilales; g__uncultured_bacterium_o_Gastranaerophilales;	516	102
291	k__Bacteria; p__Firmicutes; c__Clostridia; o__Clostridiales; f__Ruminococcaceae; g__Ruminococcus_2;	562	98
292	k__Bacteria; p__Firmicutes; c__Clostridia; o__Clostridiales; f__Lachnospiraceae; g__Acetitomaculum;	687	15
293	k__Bacteria; p__Firmicutes; c__Clostridia; o__Clostridiales; f__Lachnospiraceae; g__GCA-900066575;	701	108

294	k__Bacteria; p__Firmicutes; c__Clostridia; o__Clostridiales; f__Family_XIII; g__Family_XIII_AD3011_group;	729	310
295	k__Bacteria; p__Firmicutes; c__Clostridia; o__Clostridiales; f__Ruminococcaceae; g__Oscillibacter;	855	1541
296	k__Bacteria; p__Firmicutes; c__Negativicutes; o__Selenomonadales; f__Veillonellaceae; g__Anaerovibrio;	857	86
297	k__Bacteria; p__Firmicutes; c__Clostridia; o__Clostridiales; f__Lachnospiraceae; g__[Bacteroides]_pectinophilus_group;	971	18
298	k__Bacteria; p__Firmicutes; c__Clostridia; o__Clostridiales; f__Lachnospiraceae; g__Lachnoclostridium;	1023	264
299	k__Bacteria; p__Actinobacteria; c__Actinobacteria; o__Micrococcales; f__Micrococcaceae; g__Rothia;	1023	12466
300	k__Bacteria; p__Firmicutes; c__Clostridia; o__Clostridiales; f__Lachnospiraceae; g__Lachnospiraceae_UCG-001;	1046	414
301	k__Bacteria; p__Firmicutes; c__Clostridia; o__Clostridiales; f__Lachnospiraceae; g__Lachnospiraceae_UCG-006;	1248	204
302	k__Bacteria; p__Firmicutes; c__Clostridia; o__Clostridiales; f__Ruminococcaceae; g__Ruminiclostridium_6;	1259	102
303	k__Bacteria; p__Firmicutes; c__Clostridia; o__Clostridiales; f__Ruminococcaceae; g__Ruminococcaceae_NK4A214_group;	1332	433
304	k__Bacteria; p__Bacteroidetes; c__Bacteroidia; o__Bacteroidales; f__Bacteroidaceae; g__Bacteroides;	1482	702
305	k__Bacteria; p__Bacteroidetes; c__Bacteroidia; o__Bacteroidales; f__Prevotellaceae; g__Prevotellaceae_UCG-003;	1563	41
306	k__Bacteria; p__Firmicutes; c__Clostridia; o__Clostridiales; f__Ruminococcaceae; g__Ruminococcus_1;	1582	317

307	k__Bacteria; p__Tenericutes; c__Mollicutes; o__Mollicutes_RF39; f__uncultured_bacterium_o_Mollicutes_RF39; g__uncultured_bacterium_o_Mollicutes_RF39;	1621	251
308	k__Bacteria; p__Firmicutes; c__Clostridia; o__Clostridiales; f__Christensenellaceae; g__Christensenellaceae_R-7_group;	1683	339
309	k__Bacteria; p__Bacteroidetes; c__Bacteroidia; o__Bacteroidales; f__Prevotellaceae; g__Prevotellaceae_Ga6A1_group;	1706	46
310	k__Bacteria; p__Firmicutes; c__Negativicutes; o__Selenomonadales; f__Veillonellaceae; g__Quinella;	1825	428
311	k__Bacteria; p__Firmicutes; c__Clostridia; o__Clostridiales; f__Ruminococcaceae; g__Ruminococcaceae_UCG-005;	1876	852
312	k__Bacteria; p__Firmicutes; c__Clostridia; o__Clostridiales; f__Ruminococcaceae; g__Ruminococcaceae_UCG-003;	1891	1236
313	k__Bacteria; p__Firmicutes; c__Clostridia; o__Clostridiales; f__Lachnospiraceae; g__[Eubacterium]_ruminantium_group;	1940	29
314	k__Bacteria; p__Firmicutes; c__Erysipelotrichia; o__Erysipelotrichales; f__Erysipelotrichaceae; g__Dubosiella;	1986	399
315	k__Bacteria; p__Firmicutes; c__Clostridia; o__Clostridiales; f__Clostridiaceae_1; g__Clostridium_sensu_stricto_1;	2040	1656
316	k__Bacteria; p__Bacteroidetes; c__Bacteroidia; o__Bacteroidales; f__Rikenellaceae; g__Rikenellaceae_RC9_gut_group;	2337	620
317	k__Bacteria; p__Bacteroidetes; c__Bacteroidia; o__Bacteroidales; f__Prevotellaceae; g__Prevotellaceae_UCG-001;	2711	120
318	k__Bacteria; p__Firmicutes; c__Clostridia; o__Clostridiales; f__Ruminococcaceae; g__Ruminiclostridium_5;	2752	161
319	k__Bacteria; p__Proteobacteria; c__Deltaproteobacteria; o__Desulfovibrionales; f__Desulfovibrionaceae; g__Desulfovibrio;	2965	706

320	k__Bacteria; p__Firmicutes; c__Clostridia; o__Clostridiales; f__Lachnospiraceae; g__[Eubacterium]_xylanophilum_group;	3636	245
321	k__Bacteria; p__Firmicutes; c__Clostridia; o__Clostridiales; f__Lachnospiraceae; g__Roseburia;	4049	539
322	k__Bacteria; p__Proteobacteria; c__Deltaproteobacteria; o__Desulfovibrionales; f__Desulfovibrionaceae; g__uncultured_bacterium_f_Desulfovibrionaceae;	4799	1572
323	k__Bacteria; p__Firmicutes; c__Clostridia; o__Clostridiales; f__Ruminococcaceae; g__Ruminiclostridium_9;	5958	1819
324	k__Bacteria; p__Firmicutes; c__Clostridia; o__Clostridiales; f__Ruminococcaceae; g__uncultured_bacterium_f_Ruminococcaceae;	6620	1268
325	k__Bacteria; p__Firmicutes; c__Clostridia; o__Clostridiales; f__Ruminococcaceae; g__Ruminococcaceae_UCG-014;	6680	1264
326	k__Bacteria; p__Firmicutes; c__Clostridia; o__Clostridiales; f__Ruminococcaceae; g__[Eubacterium]_coprostanoligenes_group;	7451	1435
327	k__Bacteria; p__Firmicutes; c__Clostridia; o__Clostridiales; f__Peptostreptococcaceae; g__Romboutsia;	8231	138023
328	k__Bacteria; p__Firmicutes; c__Clostridia; o__Clostridiales; f__Ruminococcaceae; g__Ruminococcaceae_UCG-013;	8513	653
329	k__Bacteria; p__Firmicutes; c__Erysipelotrichia; o__Erysipelotrichales; f__Erysipelotrichaceae; g__Turicibacter;	8673	63554
330	k__Bacteria; p__Patescibacteria; c__Saccharimonadia; o__Saccharimonadales; f__Saccharimonadaceae; g__Candidatus_Saccharimonas;	16940	1110
331	k__Bacteria; p__Verrucomicrobia; c__Verrucomicrobiae; o__Verrucomicrobiales; f__Akkermansiaceae; g__Akkermansia;	17667	1277
332	k__Bacteria; p__Firmicutes; c__Clostridia; o__Clostridiales; f__Lachnospiraceae; g__uncultured_bacterium_f_Lachnospiraceae;	18360	2732

333	k__Bacteria; p__Spirochaetes; c__Spirochaetia; o__Spirochaetales; f__Spirochaetaceae; g__Treponema_2;	18975	300
334	k__Bacteria; p__Bacteroidetes; c__Bacteroidia; o__Bacteroidales; f__Muribaculaceae; g__uncultured_bacterium_f_Muribaculaceae;	36719	7737
335	k__Bacteria; p__Firmicutes; c__Clostridia; o__Clostridiales; f__Lachnospiraceae; g__Lachnospiraceae_NK4A136_group;	40231	8237
