# Supplementary material for: Structural and Functional Differences in Small Intestinal and Fecal Microbiota: 16S rRNA Gene Investigation in Rats
Source: Microorganisms. 2024 Aug 25;12(9):1764. doi: 10.3390/microorganisms12091764 (PMC11434385; doi:10.3390/microorganisms12091764)
Supplement: Supplementary file 1 [file microorganisms-12-01764-s001.zip › Supplementary Table 2 4 6.pdf]

Table S2. Alpha index in ileal and fecal samples of chow-diet rats (mean ± s.e.)

	n	Shannon	Simpson	Ace	Chao1	Coverage
Feces	6	4.21 ± 0.10	0.05 ± 0.01	460.59 ± 10.84	470.59 ± 13.00	0.99 ± 0.00
Ileum	6	2.92 ± 0.85	0.26 ± 0.10	606.92 ± 78.36	569.20 ± 92.76	0.99 ± 0.00

Simpson index was calculated as $\sum pi^{2}$.

Table S4. Alpha index in ileal and fecal samples of HFD/chow-diet rats (mean ± s.e.)

	n	Shannon	Simpson	Ace	Chao1	Coverage
Ileum	10	2.85 ± 0.51	0.31 ± 0.09	773.01 ± 62.06	739.30 ± 64.05	0.99 ± 0.00
Feces	10	3.81 ± 0.12	0.08 ± 0.02*	434.61 ± 8.83*	450.26 ± 11.90*	0.99 ± 0.00

*: $P < 0.05$

Table S6. Alpha index in ileal and fecal samples of SPF rats (mean ± s.e.)

	n	Shannon	Simpson	Ace	Chao1	Coverage
Ileum	6	2.64 ± 0.29	0.72 ± 0.04	955.46 ± 85.53	680.83 ± 36.54	0.99 ± 0.00
Feces	6	5.93 ± 0.23*	0.95 ± 0.01*	890.98 ± 62.72	727.66 ± 34.97	0.99 ± 0.00
