# Supplementary material for: Structural and Functional Differences in Small Intestinal and Fecal Microbiota: 16S rRNA Gene Investigation in Rats
Source: Microorganisms. 2024 Aug 25;12(9):1764. doi: 10.3390/microorganisms12091764 (PMC11434385; doi:10.3390/microorganisms12091764)
Supplement: Supplementary file 1 [file microorganisms-12-01764-s001.zip › Supplementary table 5.pdf]

Table S5. Fecal and ileal bacteria in SPF environment (*n* = 6) (genus level)

	Taxonomy	Feces	Ileum
1	k__Bacteria; p__Acidobacteria; c__Aminicenantia; o__Aminicenantales; f__uncultured_bacterium_o_Aminicenantales; g__uncultured_bacterium_o_Aminicenantales;	0	1
2	k__Bacteria; p__Acidobacteria; c__Blastocatellia_Subgroup_4; o__11-24; f__uncultured_bacterium_o_11-24; g__uncultured_bacterium_o_11-24;	0	1
3	k__Bacteria; p__Acidobacteria; c__Blastocatellia_Subgroup_4; o__Blastocatellales; f__Blastocatellaceae; g__Stenotrophobacter;	0	1
4	k__Bacteria; p__Actinobacteria; c__Acidimicrobiia; o__Microtrichales; f__Ilumatobacteraceae; g__CL500-29_marine_group;	0	1
5	k__Bacteria; p__Actinobacteria; c__Acidimicrobiia; o__Microtrichales; f__uncultured_bacterium_o_Microtrichales; g__uncultured_bacterium_o_Microtrichales;	0	1
6	k__Bacteria; p__Actinobacteria; c__Actinobacteria; o__Frankiales; f__Nakamurellaceae; g__Nakamurella;	0	1
7	k__Bacteria; p__Actinobacteria; c__Actinobacteria; o__Micrococcales; f__Micrococcaceae; g__Paenarthrobacter;	0	1
8	k__Bacteria; p__Actinobacteria; c__Actinobacteria; o__Propionibacteriales; f__Nocardioidaceae; g__Kribbella;	0	1
9	k__Bacteria; p__Actinobacteria; c__Actinobacteria; o__Pseudonocardiales; f__Pseudonocardiaceae; g__Actinophytocola;	0	1

10	k__Bacteria; p__Actinobacteria; c__Actinobacteria; o__Streptomycetales; f__Streptomycetaceae; g__Streptomyces;	0	1
11	k__Bacteria; p__Actinobacteria; c__Actinobacteria; o__Streptosporangiales; f__Streptosporangiaceae; g__Microbispora;	0	1
12	k__Bacteria; p__Actinobacteria; c__Coriobacteriia; o__Coriobacteriales; f__Eggerthellaceae; g__uncultured_bacterium_f_Eggerthellaceae;	0	1
13	k__Bacteria; p__Bacteroidetes; c__Bacteroidia; o__Bacteroidales; f__Prevotellaceae; g__Prevotellaceae_UCG-003;	0	1
14	k__Bacteria; p__Bacteroidetes; c__Bacteroidia; o__Chitinophagales; f__Chitinophagaceae; g__Taibaiella;	0	1
15	k__Bacteria; p__Bacteroidetes; c__Bacteroidia; o__uncultured_bacterium_c_Bacteroidia; f__uncultured_bacterium_c_Bacteroidia; g__uncultured_bacterium_c_Bacteroidia;	0	1
16	k__Bacteria; p__Chloroflexi; c__JG30-KF-CM66; o__uncultured_bacterium_c_JG30-KF-CM66; f__uncultured_bacterium_c_JG30-KF-CM66; g__uncultured_bacterium_c_JG30-KF-CM66;	0	1
17	k__Bacteria; p__Cyanobacteria; c__Oxyphotobacteria; o__Chloroplast; f__Desmochloris_halophila; g__Desmochloris_halophila;	0	1
18	k__Bacteria; p__Cyanobacteria; c__Oxyphotobacteria; o__Chloroplast; f__Spirogyra_maxima; g__Spirogyra_maxima;	0	1
19	k__Bacteria; p__Cyanobacteria; c__Oxyphotobacteria; o__Chloroplast; f__Trachydiscus_minutus; g__Trachydiscus_minutus;	0	1
20	k__Bacteria; p__Cyanobacteria; c__Oxyphotobacteria; o__Nostocales; f__Chroococcidiopsaceae; g__Chroococcidiopsis_SAG_2023;	0	1

21	k__Bacteria; p__Dependentiae; c__Babeliae; o__Babeliales; f__uncultured_bacterium_o_Babeliales; g__uncultured_bacterium_o_Babeliales;	0	1
22	k__Bacteria; p__Elusimicrobia; c__Endomicrobia; o__Endomicrobiales; f__Endomicrobiaceae; g__Candidatus_Endomicrobium;	0	1
23	k__Bacteria; p__Epsilonbacteraeota; c__Campylobacteria; o__Campylobacterales; f__Arcobacteraceae; g__Arcobacter;	0	1
24	k__Bacteria; p__Firmicutes; c__Bacilli; o__Bacillales; f__Staphylococcaceae; g__S31;	0	1
25	k__Bacteria; p__Firmicutes; c__Bacilli; o__Lactobacillales; f__Leuconostocaceae; g__Leuconostoc;	0	1
26	k__Bacteria; p__Firmicutes; c__Bacilli; o__Lactobacillales; f__Leuconostocaceae; g__Weissella;	0	1
27	k__Bacteria; p__Firmicutes; c__Clostridia; o__Clostridiales; f__Lachnospiraceae; g__Pseudobutyrivibrio;	0	1
28	k__Bacteria; p__Firmicutes; c__Negativicutes; o__Selenomonadales; f__Veillonellaceae; g__Pectinatus;	0	1
29	k__Bacteria; p__Fusobacteria; c__Fusobacteriia; o__Fusobacteriales; f__Fusobacteriaceae; g__Propionigenium;	0	1
30	k__Bacteria; p__Proteobacteria; c__Alphaproteobacteria; o__Puniceispirillales; f__EF100-94H03; g__uncultured_bacterium_f_EF100-94H03;	0	1
31	k__Bacteria; p__Proteobacteria; c__Alphaproteobacteria; o__Rhizobiales; f__Beijerinckiaceae; g__Psychroglaciecola;	0	1
32	k__Bacteria; p__Proteobacteria; c__Alphaproteobacteria; o__Rhizobiales; f__Methyloligellaceae; g__uncultured_bacterium_f_Methyloligellaceae;	0	1
33	k__Bacteria; p__Proteobacteria; c__Alphaproteobacteria; o__Rhizobiales; f__Rhizobiales_Incertae_Sedis; g__Bauldia;	0	1

34	k__Bacteria; p__Proteobacteria; c__Alphaproteobacteria; o__Rhizobiales; f__uncultured_bacterium_o_Rhizobiales; g__uncultured_bacterium_o_Rhizobiales;	0	1
35	k__Bacteria; p__Proteobacteria; c__Alphaproteobacteria; o__Rhodobacterales; f__Rhodobacteraceae; g__Lentibacter;	0	1
36	k__Bacteria; p__Proteobacteria; c__Deltaproteobacteria; o__Desulfobacterales; f__Desulfobacteraceae; g__Desulfococcus;	0	1
37	k__Bacteria; p__Proteobacteria; c__Deltaproteobacteria; o__Myxococcales; f__Sandaracinaceae; g__uncultured_bacterium_f_Sandaracinaceae;	0	1
38	k__Bacteria; p__Proteobacteria; c__Deltaproteobacteria; o__Oligoflexales; f__Oligoflexaceae; g__uncultured_bacterium_f_Oligoflexaceae;	0	1
39	k__Bacteria; p__Proteobacteria; c__Deltaproteobacteria; o__Syntrophobacterales; f__Syntrophaceae; g__uncultured_bacterium_f_Syntrophaceae;	0	1
40	k__Bacteria; p__Proteobacteria; c__Gammaproteobacteria; o__Aeromonadales; f__Succinivibrionaceae; g__Anaerobiospirillum;	0	1
41	k__Bacteria; p__Proteobacteria; c__Gammaproteobacteria; o__Alteromonadales; f__Shewanellaceae; g__Shewanella;	0	1
42	k__Bacteria; p__Proteobacteria; c__Gammaproteobacteria; o__Betaproteobacteriales; f__Neisseriaceae; g__uncultured_bacterium_f_Neisseriaceae;	0	1
43	k__Bacteria; p__Proteobacteria; c__Gammaproteobacteria; o__Betaproteobacteriales; f__Nitrosomonadaceae; g__DSSD61;	0	1
44	k__Bacteria; p__Proteobacteria; c__Gammaproteobacteria; o__Betaproteobacteriales; f__SC-I-84; g__beta_proteobacterium_JGI_0001003-N18;	0	1
45	k__Bacteria; p__Proteobacteria; c__Gammaproteobacteria; o__CCM19a; f__uncultured_bacterium_o_CCM19a; g__uncultured_bacterium_o_CCM19a;	0	1

46	k__Bacteria; p__Proteobacteria; c__Gammaproteobacteria; o__Enterobacteriales; f__Enterobacteriaceae; g__Rahnella;	0	1
47	k__Bacteria; p__Proteobacteria; c__Gammaproteobacteria; o__Oceanospirillales; f__Halomonadaceae; g__Salinicola;	0	1
48	k__Bacteria; p__Proteobacteria; c__Gammaproteobacteria; o__Pseudomonadales; f__Pseudomonadaceae; g__uncultured_bacterium_f_Pseudomonadaceae;	0	1
49	k__Bacteria; p__Proteobacteria; c__Gammaproteobacteria; o__R7C24; f__uncultured_bacterium_o_R7C24; g__uncultured_bacterium_o_R7C24;	0	1
50	k__Bacteria; p__Proteobacteria; c__Gammaproteobacteria; o__Salinisphaerales; f__Solimonadaceae; g__Polycyclovorans;	0	1
51	k__Bacteria; p__Proteobacteria; c__Gammaproteobacteria; o__Vibrionales; f__Vibrionaceae; g__Photobacterium;	0	1
52	k__Bacteria; p__Acidobacteria; c__Holophagae; o__Holophagales; f__Holophagaceae; g__Holophaga;	0	2
53	k__Bacteria; p__Acidobacteria; c__Subgroup_5; o__uncultured_bacterium_c_Subgroup_5; f__uncultured_bacterium_c_Subgroup_5; g__uncultured_bacterium_c_Subgroup_5;	0	2
54	k__Bacteria; p__Actinobacteria; c__Acidimicrobiia; o__Microtrichales; f__Iamiaceae; g__Iamia;	0	2
55	k__Bacteria; p__Actinobacteria; c__Actinobacteria; o__Corynebacteriales; f__Tsukamurellaceae; g__Tsukamurella;	0	2
56	k__Bacteria; p__Actinobacteria; c__Actinobacteria; o__Frankiales; f__Geodermatophilaceae; g__Geodermatophilus;	0	2
57	k__Bacteria; p__Actinobacteria; c__Actinobacteria; o__Micromonosporales; f__Micromonosporaceae; g__Micromonospora;	0	2

58	k__Bacteria; p__Actinobacteria; c__Actinobacteria; o__Micromonosporales; f__Micromonosporaceae; g__uncultured_bacterium_f_Micromonosporaceae;	0	2
59	k__Bacteria; p__Bacteroidetes; c__Bacteroidia; o__Bacteroidales; f__uncultured_bacterium_o_Bacteroidales; g__uncultured_bacterium_o_Bacteroidales;	0	2
60	k__Bacteria; p__Bacteroidetes; c__Bacteroidia; o__Cytophagales; f__Hymenobacteraceae; g__Adhaeribacter;	0	2
61	k__Bacteria; p__Chloroflexi; c__Anaerolineae; o__Anaerolineales; f__Anaerolineaceae; g__RBG-16-58-14;	0	2
62	k__Bacteria; p__Cyanobacteria; c__Oxyphotobacteria; o__Nostocales; f__Microcystaceae; g__Pleurocapsa_PCC-7327;	0	2
63	k__Bacteria; p__Firmicutes; c__Clostridia; o__Clostridiales; f__Lachnospiraceae; g__Johnsonella;	0	2
64	k__Bacteria; p__Firmicutes; c__Clostridia; o__Clostridiales; f__Lachnospiraceae; g__[Eubacterium]_ruminantium_group;	0	2
65	k__Bacteria; p__Firmicutes; c__Clostridia; o__Clostridiales; f__Peptostreptococcaceae; g__uncultured_bacterium_f_Peptostreptococcaceae;	0	2
66	k__Bacteria; p__Firmicutes; c__Clostridia; o__Clostridiales; f__Ruminococcaceae; g__DTU089;	0	2
67	k__Bacteria; p__Firmicutes; c__Negativicutes; o__Selenomonadales; f__Acidaminococcaceae; g__Succiniclasticum;	0	2
68	k__Bacteria; p__Nitrospirae; c__Nitrospira; o__Nitrospirales; f__Nitrospiraceae; g__Nitrospira;	0	2
69	k__Bacteria; p__Proteobacteria; c__Deltaproteobacteria; o__Myxococcales; f__Eel-36e1D6; g__uncultured_bacterium_f_Eel-36e1D6;	0	2
70	k__Bacteria; p__Proteobacteria; c__Deltaproteobacteria; o__Myxococcales; f__uncultured_bacterium_o_Myxococcales;	0	2

	g__uncultured_bacterium_o_Myxococcales;		
71	k__Bacteria; p__Proteobacteria; c__Deltaproteobacteria; o__Oligoflexales; f__0319-6G20; g__uncultured_bacterium_f_0319-6G20;	0	2
72	k__Bacteria; p__Proteobacteria; c__Gammaproteobacteria; o__Betaproteobacteriales; f__Burkholderiaceae; g__Polaromonas;	0	2
73	k__Bacteria; p__Acidobacteria; c__Blastocatellia_Subgroup_4; o__Blastocatellales; f__Blastocatellaceae; g__uncultured_bacterium_f_Blastocatellaceae;	0	3
74	k__Bacteria; p__Acidobacteria; c__Blastocatellia_Subgroup_4; o__Pyrinomonadales; f__Pyrinomonadaceae; g__RB41;	0	3
75	k__Bacteria; p__Actinobacteria; c__Actinobacteria; o__Frankiales; f__Geodermatophilaceae; g__Modestobacter;	0	3
76	k__Bacteria; p__Actinobacteria; c__Actinobacteria; o__Kineosporiales; f__Kineosporiaceae; g__Quadrisphaera;	0	3
77	k__Bacteria; p__Bacteroidetes; c__Bacteroidia; o__Chitinophagales; f__Chitinophagaceae; g__uncultured_bacterium_f_Chitinophagaceae;	0	3
78	k__Bacteria; p__Epsilonbacteraeota; c__Campylobacteria; o__Campylobacterales; f__Thiovulaceae; g__Sulfuricurvum;	0	3
79	k__Bacteria; p__Proteobacteria; c__Alphaproteobacteria; o__Acetobacterales; f__Acetobacteraceae; g__Roseococcus;	0	3
80	k__Bacteria; p__Proteobacteria; c__Alphaproteobacteria; o__Caulobacterales; f__Hyphomonadaceae; g__Hirschia;	0	3
81	k__Bacteria; p__Proteobacteria; c__Gammaproteobacteria; o__Betaproteobacteriales; f__Burkholderiaceae; g__Sutterella;	0	3
82	k__Bacteria; p__Firmicutes; c__Bacilli; o__Bacillales; f__Planococcaceae; g__Paenisporosarcina;	0	4

83	k__Bacteria; p__Proteobacteria; c__Alphaproteobacteria; o__Rhizobiales; f__Beijerinckiaceae; g__uncultured_bacterium_f_Beijerinckiaceae;	0	4
84	k__Bacteria; p__Proteobacteria; c__Deltaproteobacteria; o__Desulfarculales; f__Desulfarculaceae; g__uncultured_bacterium_f_Desulfarculaceae;	0	4
85	k__Bacteria; p__Firmicutes; c__Bacilli; o__Bacillales; f__Bacillaceae; g__Virgibacillus;	0	5
86	k__Bacteria; p__Proteobacteria; c__Alphaproteobacteria; o__Azospirillales; f__Azospirillaceae; g__Niveispirillum;	0	5
87	k__Bacteria; p__Proteobacteria; c__Gammaproteobacteria; o__Enterobacteriales; f__Enterobacteriaceae; g__Plesiomonas;	0	5
88	k__Bacteria; p__Proteobacteria; c__Gammaproteobacteria; o__Xanthomonadales; f__Xanthomonadaceae; g__Arenimonas;	0	5
89	k__Bacteria; p__Acidobacteria; c__Blastocatellia_Subgroup_4; o__DS-100; f__uncultured_bacterium_o_DS-100; g__uncultured_bacterium_o_DS-100;	0	6
90	k__Bacteria; p__Acidobacteria; c__Acidobacteriia; o__Acidobacteriales; f__Acidobacteriaceae_Subgroup_1; g__Edaphobacter;	1	0
91	k__Bacteria; p__Acidobacteria; c__Subgroup_6; o__uncultivated_soil_bacterium_clone_C028; f__uncultured_bacterium_o_uncultivated_soil_bacterium_clone_C028; g__uncultured_bacterium_o_uncultivated_soil_bacterium_clone_C028;	1	0
92	k__Bacteria; p__Actinobacteria; c__Acidimicrobiia; o__Actinomarinales; f__uncultured_bacterium_o_Actinomarinales; g__uncultured_bacterium_o_Actinomarinales;	1	0
93	k__Bacteria; p__Actinobacteria; c__Actinobacteria; o__Catenulisporales; f__Actinospicaceae; g__Actinospica;	1	0

94	k__Bacteria; p__Actinobacteria; c__Actinobacteria; o__Kineosporiales; f__Kineosporiaceae; g__Pseudokineococcus;	1	0
95	k__Bacteria; p__Actinobacteria; c__Actinobacteria; o__Micrococcales; f__Dermacoccaceae; g__Kytococcus;	1	0
96	k__Bacteria; p__Actinobacteria; c__Actinobacteria; o__Micrococcales; f__Promicromonosporaceae; g__Promicromonospora;	1	0
97	k__Bacteria; p__Actinobacteria; c__Actinobacteria; o__Pseudonocardiales; f__Pseudonocardiaceae; g__uncultured_bacterium_f_Pseudonocardiaceae;	1	0
98	k__Bacteria; p__Actinobacteria; c__Actinobacteria; o__Streptosporangiales; f__Nocardiopsaceae; g__Streptomonospora;	1	0
99	k__Bacteria; p__Actinobacteria; c__Thermoleophilia; o__Solirubrobacterales; f__Solirubrobacteraceae; g__Solirubrobacter;	1	0
100	k__Bacteria; p__Actinobacteria; c__Thermoleophilia; o__Solirubrobacterales; f__Solirubrobacteraceae; g__uncultured_bacterium_f_Solirubrobacteraceae;	1	0
101	k__Bacteria; p__Bacteroidetes; c__Bacteroidia; o__Bacteroidales; f__Porphyromonadaceae; g__Porphyromonas;	1	0
102	k__Bacteria; p__Bacteroidetes; c__Bacteroidia; o__Chitinophagales; f__Saprospiraceae; g__uncultured_bacterium_f_Saprospiraceae;	1	0
103	k__Bacteria; p__Chloroflexi; c__Anaerolineae; o__SBR1031; f__A4b; g__uncultured_bacterium_f_A4b;	1	0
104	k__Bacteria; p__Chloroflexi; c__Gitt-GS-136; o__uncultured_bacterium_c_Gitt-GS-136; f__uncultured_bacterium_c_Gitt-GS-136; g__uncultured_bacterium_c_Gitt-GS-136;	1	0
105	k__Bacteria; p__Cyanobacteria; c__Oxyphotobacteria; o__Synechococcales; f__Cyanobiaceae; g__Synechococcus_CC9902;	1	0

106	k__Bacteria; p__Elusimicrobia; c__Elusimicrobia; o__MVP-88; f__uncultured_bacterium_o_MVP-88; g__uncultured_bacterium_o_MVP-88;	1	0
107	k__Bacteria; p__Firmicutes; c__Bacilli; o__Bacillales; f__Bacillaceae; g__Oceanobacillus;	1	0
108	k__Bacteria; p__Firmicutes; c__Bacilli; o__Bacillales; f__Paenibacillaceae; g__Aneurinibacillus;	1	0
109	k__Bacteria; p__Firmicutes; c__Bacilli; o__Bacillales; f__Planococcaceae; g__Lysinibacillus;	1	0
110	k__Bacteria; p__Firmicutes; c__Bacilli; o__Lactobacillales; f__Carnobacteriaceae; g__Dolosigranulum;	1	0
111	k__Bacteria; p__Firmicutes; c__Bacilli; o__Lactobacillales; f__Lactobacillaceae; g__Pediococcus;	1	0
112	k__Bacteria; p__Firmicutes; c__Clostridia; o__Clostridiales; f__Christensenellaceae; g__uncultured_bacterium_f_Christensenellaceae;	1	0
113	k__Bacteria; p__Firmicutes; c__Clostridia; o__Clostridiales; f__Lachnospiraceae; g__[Eubacterium]_oxidoreducens_group;	1	0
114	k__Bacteria; p__Firmicutes; c__Negativicutes; o__Selenomonadales; f__Veillonellaceae; g__Megamonas;	1	0
115	k__Bacteria; p__Gemmatimonadetes; c__BD2-11_terrestrial_group; o__uncultured_bacterium_c_BD2-11_terrestrial_group; f__uncultured_bacterium_c_BD2-11_terrestrial_group; g__uncultured_bacterium_c_BD2-11_terrestrial_group;	1	0
116	k__Bacteria; p__Gemmatimonadetes; c__Longimicrobia; o__Longimicrobiales; f__Longimicrobiaceae; g__uncultured_bacterium_f_Longimicrobiaceae;	1	0
117	k__Bacteria; p__Proteobacteria; c__Alphaproteobacteria; o__Caulobacterales; f__Hyphomonadaceae; g__SWB02;	1	0

118	k__Bacteria; p__Proteobacteria; c__Alphaproteobacteria; o__Reyranellales; f__Reyranellaceae; g__uncultured_bacterium_f_Reyranellaceae;	1	0
119	k__Bacteria; p__Proteobacteria; c__Alphaproteobacteria; o__Rhizobiales; f__Rhizobiales_Incertae_Sedis; g__uncultured_bacterium_f_Rhizobiales_Incertae_Sedis;	1	0
120	k__Bacteria; p__Proteobacteria; c__Alphaproteobacteria; o__Rhodospirillales; f__Rhodospirillaceae; g__uncultured_bacterium_f_Rhodospirillaceae;	1	0
121	k__Bacteria; p__Proteobacteria; c__Alphaproteobacteria; o__Rickettsiales; f__Rickettsiaceae; g__Candidatus_Megaira;	1	0
122	k__Bacteria; p__Proteobacteria; c__Alphaproteobacteria; o__Sphingomonadales; f__Sphingomonadaceae; g__Croceicoccus;	1	0
123	k__Bacteria; p__Proteobacteria; c__Deltaproteobacteria; o__Bdellovibrionales; f__Bdellovibrionaceae; g__Bdellovibrio;	1	0
124	k__Bacteria; p__Proteobacteria; c__Deltaproteobacteria; o__Syntrophobacterales; f__Syntrophaceae; g__Smithella;	1	0
125	k__Bacteria; p__Proteobacteria; c__Gammaproteobacteria; o__Alteromonadales; f__Idiomarinaceae; g__Idiomarina;	1	0
126	k__Bacteria; p__Proteobacteria; c__Gammaproteobacteria; o__Betaproteobacteriales; f__Nitrosomonadaceae; g__Nitrosospira;	1	0
127	k__Bacteria; p__Proteobacteria; c__Gammaproteobacteria; o__Methylococcales; f__Methylomonaceae; g__Methylomonas;	1	0
128	k__Bacteria; p__Proteobacteria; c__Gammaproteobacteria; o__Salinisphaerales; f__Solimonadaceae; g__Nevskia;	1	0
129	k__Bacteria; p__Proteobacteria; c__Gammaproteobacteria; o__WD260; f__uncultured_bacterium_o_WD260; g__uncultured_bacterium_o_WD260;	1	0

130	k__Bacteria; p__Rokubacteria; c__NC10; o__Methylomirabilales; f__Methylomirabilaceae; g__Sh765B-TzT-35;	1	0
131	k__Bacteria; p__Acidobacteria; c__Acidobacteriia; o__Acidobacteriales; f__Acidobacteriaceae_Subgroup_1; g__Granulicella;	2	0
132	k__Bacteria; p__Acidobacteria; c__Acidobacteriia; o__Acidobacteriales; f__Acidobacteriaceae_Subgroup_1; g__Occallatibacter;	2	0
133	k__Bacteria; p__Acidobacteria; c__Subgroup_18; o__uncultured_bacterium_c_Subgroup_18; f__uncultured_bacterium_c_Subgroup_18; g__uncultured_bacterium_c_Subgroup_18;	2	0
134	k__Bacteria; p__Actinobacteria; c__Actinobacteria; o__Micrococcales; f__Demequinaceae; g__uncultured_bacterium_f_Demequinaceae;	2	0
135	k__Bacteria; p__Actinobacteria; c__Actinobacteria; o__Pseudonocardiales; f__Pseudonocardiaceae; g__Saccharomonospora;	2	0
136	k__Bacteria; p__Bacteroidetes; c__Bacteroidia; o__Bacteroidales; f__Prevotellaceae; g__Prevotella_1;	2	0
137	k__Bacteria; p__Bacteroidetes; c__Bacteroidia; o__Cytophagales; f__Spirosomaceae; g__Flectobacillus;	2	0
138	k__Bacteria; p__Bacteroidetes; c__Bacteroidia; o__Flavobacteriales; f__Weeksellaceae; g__Cloacibacterium;	2	0
139	k__Bacteria; p__Firmicutes; c__Clostridia; o__Clostridiales; f__Lachnospiraceae; g__[Eubacterium]_ventriosum_group;	2	0
140	k__Bacteria; p__Fusobacteria; c__Fusobacteriia; o__Fusobacteriales; f__Leptotrichiaceae; g__Leptotrichia;	2	0
141	k__Bacteria; p__Patescibacteria; c__Microgenomatia; o__Candidatus_Levybacteria; f__uncultured_bacterium_o_Candidatus_Levybacteria;	2	0

	g__uncultured_bacterium_o_Candidatus_Levybacteria;		
142	k__Bacteria; p__Proteobacteria; c__Alphaproteobacteria; o__Azospirillales; f__Azospirillaceae; g__Skermanella;	2	0
143	k__Bacteria; p__Proteobacteria; c__Alphaproteobacteria; o__Rhodobacterales; f__Rhodobacteraceae; g__Ascidiaceihabitans;	2	0
144	k__Bacteria; p__Proteobacteria; c__Gammaproteobacteria; o__Aeromonadales; f__Aeromonadaceae; g__Tolumonas;	2	0
145	k__Bacteria; p__Proteobacteria; c__Gammaproteobacteria; o__Xanthomonadales; f__Rhodanobacteraceae; g__Chujaibacter;	2	0
146	k__Bacteria; p__Actinobacteria; c__Actinobacteria; o__Corynebacteriales; f__Nocardiaceae; g__Gordonia;	3	0
147	k__Bacteria; p__Firmicutes; c__Clostridia; o__Clostridiales; f__Lachnospiraceae; g__[Eubacterium]_hallii_group;	3	0
148	k__Bacteria; p__Proteobacteria; c__Alphaproteobacteria; o__Azospirillales; f__uncultured_bacterium_o_Azospirillales; g__uncultured_bacterium_o_Azospirillales;	3	0
149	k__Bacteria; p__Proteobacteria; c__Alphaproteobacteria; o__Rhizobiales; f__Devosiaceae; g__Devosia;	3	0
150	k__Bacteria; p__Firmicutes; c__Clostridia; o__Clostridiales; f__Lachnospiraceae; g__Shuttleworthia;	4	0
151	k__Bacteria; p__Proteobacteria; c__Alphaproteobacteria; o__Rhodobacterales; f__Rhodobacteraceae; g__Gemmobacter;	4	0
152	k__Bacteria; p__Proteobacteria; c__Gammaproteobacteria; o__Xanthomonadales; f__Xanthomonadaceae; g__uncultured_bacterium_f_Xanthomonadaceae;	4	0

153	k__Bacteria; p__Actinobacteria; c__Actinobacteria; o__Micrococcales; f__Promicromonosporaceae; g__Cellulosimicrobium;	13	0
154	k__Bacteria; p__Elusimicrobia; c__Elusimicrobia; o__Elusimicrobiales; f__Elusimicrobiaceae; g__Elusimicrobium;	39	0
155	k__Bacteria; p__Firmicutes; c__Clostridia; o__Clostridiales; f__Ruminococcaceae; g__Negativibacillus;	57	0
156	k__Bacteria; p__Firmicutes; c__Clostridia; o__Clostridiales; f__Ruminococcaceae; g__Ruminococcaceae_UCG-010;	59	0
157	k__Bacteria; p__Firmicutes; c__Clostridia; o__Clostridiales; f__Ruminococcaceae; g__Ruminococcaceae_UCG-009;	81	0
158	k__Bacteria; p__Firmicutes; c__Clostridia; o__Clostridiales; f__Ruminococcaceae; g__Ruminococcaceae_UCG-004;	103	0
159	k__Bacteria; p__Firmicutes; c__Clostridia; o__Clostridiales; f__Ruminococcaceae; g__Pygmaiobacter;	231	0
160	k__Bacteria; p__Acetothermia; c__Acetothermiia; o__uncultured_bacterium_c_Acetothermiia; f__uncultured_bacterium_c_Acetothermiia; g__uncultured_bacterium_c_Acetothermiia;	1	1
161	k__Bacteria; p__Acidobacteria; c__Acidobacteriia; o__Acidobacteriales; f__Acidobacteriaceae_Subgroup_1; g__Acidipila;	1	1
162	k__Bacteria; p__Actinobacteria; c__Actinobacteria; o__Micrococcales; f__Dermabacteraceae; g__Dermabacter;	1	1
163	k__Bacteria; p__Actinobacteria; c__Actinobacteria; o__Streptosporangiales; f__Nocardiopsaceae; g__Nocardiopsis;	1	1
164	k__Bacteria; p__Bacteroidetes; c__Bacteroidia; o__Cytophagales; f__Hymenobacteraceae; g__Hymenobacter;	1	1

165	k__Bacteria; p__Bacteroidetes; c__Bacteroidia; o__Cytophagales; f__Microscillaceae; g__Chryseolinea;	1	1
166	k__Bacteria; p__Bacteroidetes; c__Bacteroidia; o__Cytophagales; f__Spirosomaceae; g__Persicitalea;	1	1
167	k__Bacteria; p__Firmicutes; c__Bacilli; o__Lactobacillales; f__Carnobacteriaceae; g__Trichococcus;	1	1
168	k__Bacteria; p__Firmicutes; c__Clostridia; o__Clostridiales; f__Family_XI; g__Finegoldia;	1	1
169	k__Bacteria; p__Firmicutes; c__Clostridia; o__Clostridiales; f__Lachnospiraceae; g__Coprococcus_2;	1	1
170	k__Bacteria; p__Firmicutes; c__Clostridia; o__Clostridiales; f__Peptostreptococcaceae; g__Terrisporobacter;	1	1
171	k__Bacteria; p__Firmicutes; c__Negativicutes; o__Selenomonadales; f__Acidaminococcaceae; g__Phascolarctobacterium;	1	1
172	k__Bacteria; p__Gemmatimonadetes; c__Gemmatimonadetes; o__Gemmatimonadales; f__Gemmatimonadaceae; g__Gemmatirosa;	1	1
173	k__Bacteria; p__Nitrospirae; c__Thermodesulfovibrionia; o__uncultured_bacterium_c_Thermodesulfovibrionia; f__uncultured_bacterium_c_Thermodesulfovibrionia; g__uncultured_bacterium_c_Thermodesulfovibrionia;	1	1
174	k__Bacteria; p__Proteobacteria; c__Alphaproteobacteria; o__Elsterales; f__URHD0088; g__uncultured_bacterium_f_URHD0088;	1	1
175	k__Bacteria; p__Proteobacteria; c__Alphaproteobacteria; o__Rhizobiales; f__Hyphomicrobiaceae; g__uncultured_bacterium_f_Hyphomicrobiaceae;	1	1
176	k__Bacteria; p__Proteobacteria; c__Alphaproteobacteria; o__Rhodobacterales; f__Rhodobacteraceae; g__Amaricoccus;	1	1

177	k__Bacteria; p__Proteobacteria; c__Alphaproteobacteria; o__Rhodobacterales; f__Rhodobacteraceae; g__Roseobacter_clade_CHAB-I-5_lineage;	1	1
178	k__Bacteria; p__Proteobacteria; c__Deltaproteobacteria; o__Myxococcales; f__bacteriap25; g__uncultured_bacterium_f_bacteriap25;	1	1
179	k__Bacteria; p__Proteobacteria; c__Gammaproteobacteria; o__Betaproteobacteriales; f__Burkholderiaceae; g__Acidovorax;	1	1
180	k__Bacteria; p__Proteobacteria; c__Gammaproteobacteria; o__Betaproteobacteriales; f__Burkholderiaceae; g__Ramlibacter;	1	1
181	k__Bacteria; p__Proteobacteria; c__Gammaproteobacteria; o__Betaproteobacteriales; f__Hydrogenophilaceae; g__Thiobacillus;	1	1
182	k__Bacteria; p__Proteobacteria; c__Gammaproteobacteria; o__Diplorickettsiales; f__Diplorickettsiaceae; g__uncultured_bacterium_f_Diplorickettsiaceae;	1	1
183	k__Bacteria; p__Proteobacteria; c__Gammaproteobacteria; o__KF-JG30-C25; f__uncultured_bacterium_o_KF-JG30-C25; g__uncultured_bacterium_o_KF-JG30-C25;	1	1
184	k__Bacteria; p__Proteobacteria; c__Gammaproteobacteria; o__Xanthomonadales; f__Xanthomonadaceae; g__Luteimonas;	1	1
185	k__Bacteria; p__Proteobacteria; c__Gammaproteobacteria; o__Xanthomonadales; f__Xanthomonadaceae; g__Silanimonas;	1	1
186	k__Bacteria; p__Actinobacteria; c__Actinobacteria; o__Micrococcales; f__Cellulomonadaceae; g__Cellulomonas;	2	1
187	k__Bacteria; p__Actinobacteria; c__Actinobacteria; o__Pseudonocardiales; f__Pseudonocardiaceae; g__Amycolatopsis;	2	1
188	k__Bacteria; p__Actinobacteria; c__Thermoleophilia; o__Solirubrobacterales; f__67-14; g__uncultured_bacterium_f_67-14;	2	1

189	k__Bacteria; p__Bacteroidetes; c__Bacteroidia; o__Cytophagales; f__Microscillaceae; g__uncultured_bacterium_f_Microscillaceae;	2	1
190	k__Bacteria; p__Chloroflexi; c__Ktedonobacteria; o__Ktedonobacterales; f__JG30-KF-AS9; g__uncultured_bacterium_f_JG30-KF-AS9;	2	1
191	k__Bacteria; p__Cyanobacteria; c__Oxyphotobacteria; o__Nostocales; f__Chroococcidiopsaceae; g__Aliterella_CENA595;	2	1
192	k__Bacteria; p__Firmicutes; c__Bacilli; o__Bacillales; f__Planococcaceae; g__Kurthia;	2	1
193	k__Bacteria; p__Firmicutes; c__Clostridia; o__Clostridiales; f__Ruminococcaceae; g__Butyricicoccus;	2	1
194	k__Bacteria; p__Proteobacteria; c__Alphaproteobacteria; o__Rhizobiales; f__KF-JG30-B3; g__uncultured_bacterium_f_KF-JG30-B3;	2	1
195	k__Bacteria; p__Proteobacteria; c__Alphaproteobacteria; o__Rhodobacterales; f__Rhodobacteraceae; g__Rhodobacter;	2	1
196	k__Bacteria; p__Proteobacteria; c__Alphaproteobacteria; o__Sphingomonadales; f__Sphingomonadaceae; g__Altererythrobacter;	2	1
197	k__Bacteria; p__Proteobacteria; c__Deltaproteobacteria; o__Deltaproteobacteria_Incertae_Sedis; f__Syntrophorhabdaceae; g__Syntrophorhabdus;	2	1
198	k__Bacteria; p__Proteobacteria; c__Gammaproteobacteria; o__Betaproteobacteriales; f__Methylophilaceae; g__Methylotenera;	2	1
199	k__Bacteria; p__Rokubacteria; c__NC10; o__Rokubacteriales; f__bacterium_WX65; g__uncultured_bacterium_f_bacterium_WX65;	2	1
200	k__Bacteria; p__Actinobacteria; c__Actinobacteria; o__Propionibacteriales; f__Nocardioidaceae; g__Nocardioides;	3	1
201	k__Bacteria; p__Actinobacteria; c__Thermoleophilia; o__Gaiellales; f__Gaiellaceae; g__Gaiella;	3	1

202	k__Bacteria; p__Firmicutes; c__Bacilli; o__Bacillales; f__Bacillaceae; g__uncultured_bacterium_f_Bacillaceae;	3	1
203	k__Bacteria; p__Firmicutes; c__Clostridia; o__Clostridiales; f__Clostridiaceae_1; g__Clostridium_sensu_stricto_11;	3	1
204	k__Bacteria; p__Firmicutes; c__Clostridia; o__Clostridiales; f__Ruminococcaceae; g__Flavonifractor;	3	1
205	k__Bacteria; p__Firmicutes; c__Negativicutes; o__Selenomonadales; f__Veillonellaceae; g__uncultured_bacterium_f_Veillonellaceae;	3	1
206	k__Bacteria; p__Proteobacteria; c__Alphaproteobacteria; o__Acetobacterales; f__Acetobacteraceae; g__Rubritepida;	3	1
207	k__Bacteria; p__Proteobacteria; c__Alphaproteobacteria; o__Dongiales; f__Dongiaceae; g__Dongia;	4	1
208	k__Bacteria; p__Proteobacteria; c__Gammaproteobacteria; o__Betaproteobacteriales; f__Rhodocyclaceae; g__uncultured_bacterium_f_Rhodocyclaceae;	4	1
209	k__Bacteria; p__Proteobacteria; c__Gammaproteobacteria; o__Betaproteobacteriales; f__Methylophilaceae; g__Methylophilus;	6	1
210	k__Bacteria; p__Firmicutes; c__Clostridia; o__Clostridiales; f__Lachnospiraceae; g__Lachnospiraceae_FCS020_group;	40	1
211	k__Bacteria; p__Firmicutes; c__Clostridia; o__Clostridiales; f__Ruminococcaceae; g__Intestinimonas;	102	1
212	k__Bacteria; p__Acidobacteria; c__Acidobacteriia; o__Solibacterales; f__Solibacteraceae_Subgroup_3; g__uncultured_bacterium_f_Solibacteraceae_Subgroup_3;	1	2
213	k__Bacteria; p__Actinobacteria; c__Actinobacteria; o__Frankiales; f__Frankiaceae; g__Jatrophihabitans;	1	2

214	k__Bacteria; p__Actinobacteria; c__Actinobacteria; o__Frankiales; f__uncultured_bacterium_o_Frankiales; g__uncultured_bacterium_o_Frankiales;	1	2
215	k__Bacteria; p__Bacteroidetes; c__Bacteroidia; o__Bacteroidales; f__Bacteroidetes_vadinHA17; g__uncultured_bacterium_f_Bacteroidetes_vadinHA17;	1	2
216	k__Bacteria; p__Caldiserica; c__Caldisericia; o__Caldisericales; f__Caldisericaceae; g__Caldisericum;	1	2
217	k__Bacteria; p__Epsilonbacteraeota; c__Campylobacteria; o__Campylobacterales; f__Thiovulaceae; g__Sulfurimonas;	1	2
218	k__Bacteria; p__Gemmatimonadetes; c__S0134_terrestrial_group; o__uncultured_bacterium_c_S0134_terrestrial_group; f__uncultured_bacterium_c_S0134_terrestrial_group; g__uncultured_bacterium_c_S0134_terrestrial_group;	1	2
219	k__Bacteria; p__Proteobacteria; c__Alphaproteobacteria; o__Azospirillales; f__Azospirillaceae; g__Azospirillum;	1	2
220	k__Bacteria; p__Proteobacteria; c__Alphaproteobacteria; o__Rhizobiales; f__A0839; g__uncultured_bacterium_f_A0839;	1	2
221	k__Bacteria; p__Proteobacteria; c__Alphaproteobacteria; o__Rhizobiales; f__Rhizobiaceae; g__Chelativorans;	1	2
222	k__Bacteria; p__Proteobacteria; c__Deltaproteobacteria; o__Myxococcales; f__BIrii41; g__uncultured_bacterium_f_BIrii41;	1	2
223	k__Bacteria; p__Proteobacteria; c__Gammaproteobacteria; o__Alteromonadales; f__Alteromonadaceae; g__Rheinheimera;	1	2
224	k__Bacteria; p__Proteobacteria; c__Gammaproteobacteria; o__Enterobacteriales; f__Enterobacteriaceae; g__Pectobacterium;	1	2
225	k__Bacteria; p__Actinobacteria; c__Acidimicrobiia; o__IMCC26256; f__actinobacterium_BGR_88; g__uncultured_bacterium_f_actinobacterium_BGR_88;	2	2

226	k__Bacteria; p__Actinobacteria; c__Acidimicrobiia; o__IMCC26256; f__bacterium_enrichment_culture_clone_auto73_4W; g__uncultured_bacterium_f_bacterium_enrichment_culture_clone_auto73_4W;	2	2
227	k__Bacteria; p__Actinobacteria; c__Actinobacteria; o__Catenulisporales; f__Catenulisporaceae; g__Catenulispora;	2	2
228	k__Bacteria; p__Deinococcus-Thermus; c__Deinococci; o__Deinococcales; f__Trueperaceae; g__Truepera;	2	2
229	k__Bacteria; p__Epsilonbacteraeota; c__Campylobacteria; o__Campylobacterales; f__Campylobacteraceae; g__Campylobacter;	2	2
230	k__Bacteria; p__Firmicutes; c__Bacilli; o__Bacillales; f__Bacillaceae; g__Aeribacillus;	2	2
231	k__Bacteria; p__Firmicutes; c__Bacilli; o__Bacillales; f__Planococcaceae; g__Domibacillus;	2	2
232	k__Bacteria; p__Proteobacteria; c__Alphaproteobacteria; o__Rhodobacterales; f__Rhodobacteraceae; g__Rubellimicrobium;	2	2
233	k__Bacteria; p__Proteobacteria; c__Gammaproteobacteria; o__Betaproteobacteriales; f__Burkholderiaceae; g__Curvibacter;	2	2
234	k__Bacteria; p__Proteobacteria; c__Gammaproteobacteria; o__SZB30; f__uncultured_bacterium_o_SZB30; g__uncultured_bacterium_o_SZB30;	2	2
235	k__Bacteria; p__Bacteroidetes; c__Rhodothermia; o__Balneolales; f__Balneolaceae; g__uncultured_bacterium_f_Balneolaceae;	3	2
236	k__Bacteria; p__Cyanobacteria; c__Oxyphotobacteria; o__Nostocales; f__uncultured_bacterium_o_Nostocales; g__uncultured_bacterium_o_Nostocales;	3	2
237	k__Bacteria; p__Firmicutes; c__Clostridia; o__Clostridiales; f__Peptostreptococcaceae; g__Intestinibacter;	3	2

238	k__Bacteria; p__Proteobacteria; c__Alphaproteobacteria; o__Tistrellales; f__Geminicoccaceae; g__uncultured_bacterium_f_Geminicoccaceae;	3	2
239	k__Bacteria; p__Proteobacteria; c__Gammaproteobacteria; o__Betaproteobacteriales; f__Burkholderiaceae; g__Lautropia;	3	2
240	k__Bacteria; p__Proteobacteria; c__Gammaproteobacteria; o__Betaproteobacteriales; f__Burkholderiaceae; g__Pseudoduganella;	3	2
241	k__Bacteria; p__Proteobacteria; c__Gammaproteobacteria; o__Xanthomonadales; f__Rhodanobacteraceae; g__uncultured_bacterium_f_Rhodanobacteraceae;	3	2
242	k__Bacteria; p__uncultured_bacterium_k_Bacteria; c__uncultured_bacterium_k_Bacteria; o__uncultured_bacterium_k_Bacteria; f__uncultured_bacterium_k_Bacteria; g__uncultured_bacterium_k_Bacteria;	3	2
243	Unassigned; Unassigned; Unassigned; Unassigned; Unassigned; Unassigned;	4	2
244	k__Bacteria; p__Firmicutes; c__Bacilli; o__Bacillales; f__Paenibacillaceae; g__Brevibacillus;	4	2
245	k__Bacteria; p__Proteobacteria; c__Alphaproteobacteria; o__Rhizobiales; f__Beijerinckiaceae; g__Bosea;	4	2
246	k__Bacteria; p__Proteobacteria; c__Gammaproteobacteria; o__Betaproteobacteriales; f__Burkholderiaceae; g__Pelomonas;	4	2
247	k__Bacteria; p__WPS-2; c__uncultured_bacterium_p_WPS-2; o__uncultured_bacterium_p_WPS-2; f__uncultured_bacterium_p_WPS-2; g__uncultured_bacterium_p_WPS-2;	4	2

248	k__Bacteria; p__Acidobacteria; c__Acidobacteriia; o__Solibacterales; f__Solibacteraceae_Subgroup_3; g__Paludibaculum;	5	2
249	k__Bacteria; p__Acidobacteria; c__Subgroup_6; o__Acidobacteria_bacterium_IGE-011; f__uncultured_bacterium_o_Acidobacteria_bacterium_IGE-011; g__uncultured_bacterium_o_Acidobacteria_bacterium_IGE-011;	5	2
250	k__Bacteria; p__Chloroflexi; c__Anaerolineae; o__Anaerolineales; f__Anaerolineaceae; g__uncultured_bacterium_f_Anaerolineaceae;	6	2
251	k__Bacteria; p__Proteobacteria; c__Alphaproteobacteria; o__Caulobacterales; f__Parvularculaceae; g__Amphiplicatus;	6	2
252	k__Bacteria; p__Firmicutes; c__Clostridia; o__Clostridiales; f__Lachnospiraceae; g__Tyzzerella_4;	26	2
253	k__Bacteria; p__Firmicutes; c__Clostridia; o__Clostridiales; f__Lachnospiraceae; g__Lachnospiraceae_UCG-001;	43	2
254	k__Bacteria; p__Firmicutes; c__Clostridia; o__Clostridiales; f__Family_XIII; g__Family_XIII_UCG-001;	130	2
255	k__Bacteria; p__Firmicutes; c__Clostridia; o__Clostridiales; f__Family_XIII; g__uncultured_bacterium_f_Family_XIII;	140	2
256	k__Bacteria; p__Tenericutes; c__Mollicutes; o__Anaeroplasmatales; f__Anaeroplasmataceae; g__Anaeroplasma;	249	2
257	k__Bacteria; p__Firmicutes; c__Clostridia; o__Clostridiales; f__Lachnospiraceae; g__Tyzzerella;	628	2
258	k__Bacteria; p__Actinobacteria; c__Acidimicrobiia; o__Microtrichales; f__Ilumatobacteraceae; g__Ilumatobacter;	1	3
259	k__Bacteria; p__Actinobacteria; c__Actinobacteria; o__Corynebacteriales; f__Corynebacteriaceae; g__Corynebacterium;	1	3

260	k__Bacteria; p__Firmicutes; c__Clostridia; o__Clostridiales; f__Lachnospiraceae; g__Tyzzerella_3;	1	3
261	k__Bacteria; p__Proteobacteria; c__Alphaproteobacteria; o__Acetobacterales; f__Acetobacteraceae; g__Acidicaldus;	1	3
262	k__Bacteria; p__Proteobacteria; c__Deltaproteobacteria; o__Myxococcales; f__Polyangiaceae; g__Polyangium;	1	3
263	k__Bacteria; p__Actinobacteria; c__Coriobacteriia; o__Coriobacteriales; f__Atopobiaceae; g__uncultured_bacterium_f_Atopobiaceae;	2	3
264	k__Bacteria; p__Firmicutes; c__Bacilli; o__Bacillales; f__Family_XII; g__Exiguobacterium;	2	3
265	k__Bacteria; p__Proteobacteria; c__Alphaproteobacteria; o__Rhizobiales; f__Rhizobiales_Incertae_Sedis; g__Nordella;	2	3
266	k__Bacteria; p__Proteobacteria; c__Gammaproteobacteria; o__Enterobacteriales; f__Enterobacteriaceae; g__Pantoea;	2	3
267	k__Bacteria; p__Actinobacteria; c__Actinobacteria; o__Micrococcales; f__Microbacteriaceae; g__Microbacterium;	3	3
268	k__Bacteria; p__Actinobacteria; c__Thermoleophilia; o__Gaiellales; f__uncultured_bacterium_o_Gaiellales; g__uncultured_bacterium_o_Gaiellales;	3	3
269	k__Bacteria; p__Proteobacteria; c__Alphaproteobacteria; o__Rhizobiales; f__Stappiaceae; g__uncultured_bacterium_f_Stappiaceae;	3	3
270	k__Bacteria; p__Acidobacteria; c__Subgroup_17; o__uncultured_bacterium_c_Subgroup_17; f__uncultured_bacterium_c_Subgroup_17; g__uncultured_bacterium_c_Subgroup_17;	4	3
271	k__Bacteria; p__Firmicutes; c__Clostridia; o__Clostridiales; f__Lachnospiraceae; g__[Eubacterium]_eligens_group;	4	3

272	k__Bacteria; p__Proteobacteria; c__Alphaproteobacteria; o__Rhizobiales; f__Hyphomicrobiaceae; g__Pedomicrobium;	4	3
273	k__Bacteria; p__Actinobacteria; c__Acidimicrobiia; o__uncultured_bacterium_c_Acidimicrobiia; f__uncultured_bacterium_c_Acidimicrobiia; g__uncultured_bacterium_c_Acidimicrobiia;	5	3
274	k__Bacteria; p__Actinobacteria; c__Actinobacteria; o__Corynebacteriales; f__Corynebacteriaceae; g__Lawsonella;	5	3
275	k__Bacteria; p__Proteobacteria; c__Gammaproteobacteria; o__Vibrionales; f__Vibrionaceae; g__Vibrio;	5	3
276	k__Bacteria; p__Proteobacteria; c__Gammaproteobacteria; o__Xanthomonadales; f__Rhodanobacteraceae; g__Rhodanobacter;	5	3
277	k__Bacteria; p__Firmicutes; c__Clostridia; o__Clostridiales; f__Family_XI; g__Peptoniphilus;	6	3
278	k__Bacteria; p__Proteobacteria; c__Alphaproteobacteria; o__Rhizobiales; f__Hyphomicrobiaceae; g__Hyphomicrobium;	6	3
279	k__Bacteria; p__Firmicutes; c__Clostridia; o__Clostridiales; f__Family_XIII; g__[Eubacterium]_nodatum_group;	75	3
280	k__Bacteria; p__Firmicutes; c__Clostridia; o__Clostridiales; f__Lachnospiraceae; g__GCA-900066575;	176	3
281	k__Bacteria; p__Actinobacteria; c__Acidimicrobiia; o__Microtrichales; f__Ilumatobacteraceae; g__uncultured_bacterium_f_Ilumatobacteraceae;	1	4
282	k__Bacteria; p__Chloroflexi; c__KD4-96; o__uncultured_bacterium_c_KD4-96; f__uncultured_bacterium_c_KD4-96; g__uncultured_bacterium_c_KD4-96;	1	4

283	k__Bacteria; p__Firmicutes; c__Bacilli; o__Bacillales; f__Alicyclobacillaceae; g__Tumebacillus;	1	4
284	k__Bacteria; p__Patescibacteria; c__Saccharimonadia; o__Saccharimonadales; f__Saccharimonadaceae; g__uncultured_bacterium_f_Saccharimonadaceae;	1	4
285	k__Bacteria; p__Proteobacteria; c__Deltaproteobacteria; o__SAR324_cladeMarine_group_B; f__bacterium_enrichment_culture_clone_B302011; g__uncultured_bacterium_f_bacterium_enrichment_culture_clone_B302011;	1	4
286	k__Bacteria; p__Patescibacteria; c__Saccharimonadia; o__Saccharimonadales; f__uncultured_bacterium_o_Saccharimonadales; g__uncultured_bacterium_o_Saccharimonadales;	2	4
287	k__Bacteria; p__Proteobacteria; c__Alphaproteobacteria; o__Rhizobiales; f__Rhizobiaceae; g__uncultured_bacterium_f_Rhizobiaceae;	2	4
288	k__Bacteria; p__Proteobacteria; c__Gammaproteobacteria; o__CCD24; f__uncultured_bacterium_o_CCD24; g__uncultured_bacterium_o_CCD24;	2	4
289	k__Bacteria; p__Actinobacteria; c__Acidimicrobiia; o__IMCC26256; f__uncultured_bacterium_o_IMCC26256; g__uncultured_bacterium_o_IMCC26256;	3	4
290	k__Bacteria; p__Proteobacteria; c__Alphaproteobacteria; o__Sphingomonadales; f__Sphingomonadaceae; g__Sphingopyxis;	3	4
291	k__Bacteria; p__Actinobacteria; c__Actinobacteria; o__Corynebacteriales; f__Mycobacteriaceae; g__Mycobacterium;	4	4
292	k__Bacteria; p__Actinobacteria; c__Actinobacteria; o__Micrococcales; f__Micrococcaceae; g__Arthrobacter;	4	4
293	k__Bacteria; p__Fusobacteria; c__Fusobacteriia; o__Fusobacteriales; f__Fusobacteriaceae; g__Cetobacterium;	5	4

294	k__Bacteria; p__Proteobacteria; c__Alphaproteobacteria; o__Rhodobacterales; f__Rhodobacteraceae; g__uncultured_bacterium_f_Rhodobacteraceae;	5	4
295	k__Bacteria; p__Proteobacteria; c__Alphaproteobacteria; o__Sphingomonadales; f__Sphingomonadaceae; g__DSSF69;	5	4
296	k__Bacteria; p__Proteobacteria; c__Alphaproteobacteria; o__Sphingomonadales; f__Sphingomonadaceae; g__Erythrobacter;	5	4
297	k__Bacteria; p__Proteobacteria; c__Gammaproteobacteria; o__Betaproteobacteriales; f__Rhodocyclaceae; g__Dechloromonas;	5	4
298	k__Bacteria; p__Acidobacteria; c__Subgroup_6; o__uncultivated_soil_bacterium_clone_C112; f__uncultured_bacterium_o_uncultivated_soil_bacterium_clone_C112; g__uncultured_bacterium_o_uncultivated_soil_bacterium_clone_C112;	6	4
299	k__Bacteria; p__Actinobacteria; c__Actinobacteria; o__Micrococcales; f__Bogoriellaceae; g__uncultured_bacterium_f_Bogoriellaceae;	7	4
300	k__Bacteria; p__Proteobacteria; c__Gammaproteobacteria; o__Betaproteobacteriales; f__Nitrosomonadaceae; g__Ellin6067;	7	4
301	k__Bacteria; p__Actinobacteria; c__Coriobacteriia; o__Coriobacteriales; f__Coriobacteriaceae; g__Collinsella;	10	4
302	k__Bacteria; p__Firmicutes; c__Clostridia; o__Clostridiales; f__Clostridiales_vadinBB60_group; g__uncultured_bacterium_f_Clostridiales_vadinBB60_group;	23	4
303	k__Bacteria; p__Firmicutes; c__Clostridia; o__Clostridiales; f__Ruminococcaceae; g__Ruminiclostridium;	288	4
304	k__Bacteria; p__Bacteroidetes; c__Bacteroidia; o__Cytophagales; f__Spirosomaceae; g__Dyadobacter;	1	5

305	k__Bacteria; p__Proteobacteria; c__Alphaproteobacteria; o__Rhodospirillales; f__uncultured_bacterium_o_Rhodospirillales; g__uncultured_bacterium_o_Rhodospirillales;	1	5
306	k__Bacteria; p__Proteobacteria; c__Gammaproteobacteria; o__Nitrococcales; f__Nitrococcales_Incertae_Sedis; g__Methylonatrum;	1	5
307	k__Bacteria; p__Proteobacteria; c__Gammaproteobacteria; o__Steroidobacterales; f__Steroidobacteraceae; g__Steroidobacter;	1	5
308	k__Bacteria; p__Acidobacteria; c__Thermoanaerobaculia; o__Thermoanaerobaculales; f__Thermoanaerobaculaceae; g__Subgroup_10;	2	5
309	k__Bacteria; p__Firmicutes; c__Bacilli; o__Bacillales; f__Paenibacillaceae; g__Paenibacillus;	2	5
310	k__Bacteria; p__Fusobacteria; c__Fusobacteriia; o__Fusobacteriales; f__Fusobacteriaceae; g__Fusobacterium;	2	5
311	k__Bacteria; p__Proteobacteria; c__Deltaproteobacteria; o__Myxococcales; f__Polyangiaceae; g__Pajaroellobacter;	2	5
312	k__Bacteria; p__Proteobacteria; c__Gammaproteobacteria; o__PLTA13; f__uncultured_bacterium_o_PLTA13; g__uncultured_bacterium_o_PLTA13;	2	5
313	k__Bacteria; p__Actinobacteria; c__Actinobacteria; o__Frankiales; f__Geodermatophilaceae; g__Blastococcus;	3	5
314	k__Bacteria; p__Proteobacteria; c__Alphaproteobacteria; o__Micropepsales; f__Micropepsaceae; g__uncultured_bacterium_f_Micropepsaceae;	3	5
315	k__Bacteria; p__Proteobacteria; c__Gammaproteobacteria; o__Betaproteobacteriales; f__Burkholderiaceae; g__Rhizobacter;	3	5
316	k__Bacteria; p__Firmicutes; c__Clostridia; o__Clostridiales; f__Lachnospiraceae; g__Lachnospiraceae_UCG-004;	4	5

317	k__Bacteria; p__Proteobacteria; c__Deltaproteobacteria; o__uncultured_bacterium_c_Deltaproteobacteria; f__uncultured_bacterium_c_Deltaproteobacteria; g__uncultured_bacterium_c_Deltaproteobacteria;	4	5
318	k__Bacteria; p__Proteobacteria; c__Gammaproteobacteria; o__Betaproteobacteriales; f__Burkholderiaceae; g__Aquabacterium;	5	5
319	k__Bacteria; p__Proteobacteria; c__Gammaproteobacteria; o__Xanthomonadales; f__Xanthomonadaceae; g__Pseudoxanthomonas;	6	5
320	k__Bacteria; p__Proteobacteria; c__Alphaproteobacteria; o__Sphingomonadales; f__Sphingomonadaceae; g__Sphingobium;	9	5
321	k__Bacteria; p__Proteobacteria; c__Gammaproteobacteria; o__Pasteurellales; f__Pasteurellaceae; g__Haemophilus;	10	5
322	k__Bacteria; p__Firmicutes; c__Clostridia; o__Clostridiales; f__Ruminococcaceae; g__Ruminococcus_2;	46	5
323	k__Bacteria; p__Proteobacteria; c__Gammaproteobacteria; o__Aeromonadales; f__Aeromonadaceae; g__Aeromonas;	161	5
324	k__Bacteria; p__Firmicutes; c__Clostridia; o__Clostridiales; f__uncultured_bacterium_o_Clostridiales; g__uncultured_bacterium_o_Clostridiales;	225	5
325	k__Bacteria; p__Actinobacteria; c__MB-A2-108; o__uncultured_bacterium_c_MB-A2-108; f__uncultured_bacterium_c_MB-A2-108; g__uncultured_bacterium_c_MB-A2-108;	2	6
326	k__Bacteria; p__Bacteroidetes; c__Bacteroidia; o__Chitinophagales; f__Chitinophagaceae; g__Sediminibacterium;	2	6
327	k__Bacteria; p__Proteobacteria; c__Gammaproteobacteria; o__Betaproteobacteriales; f__SC-I-84; g__uncultured_bacterium_f_SC-I-84;	2	6

328	k__Bacteria; p__Proteobacteria; c__Gammaproteobacteria; o__Xanthomonadales; f__Xanthomonadaceae; g__Lysobacter;	2	6
329	k__Bacteria; p__Proteobacteria; c__Deltaproteobacteria; o__Myxococcales; f__Haliangiaceae; g__Haliangium;	5	6
330	k__Bacteria; p__Verrucomicrobia; c__Verrucomicrobiae; o__Chthoniobacterales; f__Chthoniobacteraceae; g__Candidatus_Udaeobacter;	5	6
331	k__Bacteria; p__Proteobacteria; c__Gammaproteobacteria; o__Betaproteobacteriales; f__Burkholderiaceae; g__Candidatus_Vidania;	6	6
332	k__Bacteria; p__Proteobacteria; c__Alphaproteobacteria; o__Rhodobacterales; f__Rhodobacteraceae; g__Paracoccus;	7	6
333	k__Bacteria; p__Proteobacteria; c__Gammaproteobacteria; o__Betaproteobacteriales; f__TRA3-20; g__uncultured_bacterium_f_TRA3-20;	7	6
334	k__Bacteria; p__Proteobacteria; c__Alphaproteobacteria; o__Caulobacterales; f__Caulobacteraceae; g__Phenylobacterium;	8	6
335	k__Bacteria; p__Firmicutes; c__Clostridia; o__Clostridiales; f__Lachnospiraceae; g__[Ruminococcus]_torques_group;	14	6
336	k__Bacteria; p__Firmicutes; c__Clostridia; o__Clostridiales; f__Lachnospiraceae; g__Lachnospiraceae_UCG-010;	136	6
337	k__Bacteria; p__Firmicutes; c__Erysipelotrichia; o__Erysipelotrichales; f__Erysipelotrichaceae; g__Candidatus_Stoquefichus;	282	6
338	k__Bacteria; p__Proteobacteria; c__Gammaproteobacteria; o__Betaproteobacteriales; f__Rhodocyclaceae; g__Methyloversatilis;	2	7
339	k__Bacteria; p__Firmicutes; c__Bacilli; o__Lactobacillales; f__uncultured_bacterium_o_Lactobacillales; g__uncultured_bacterium_o_Lactobacillales;	3	7
340	k__Bacteria; p__Acidobacteria; c__Acidobacteriia; o__Acidobacteriales; f__Acidobacteriaceae_Subgroup_1;	4	7

	g__uncultured_bacterium_f_Acidobacteriaceae_Subgroup_1;		
341	k__Bacteria; p__Acidobacteria; c__Holophagae; o__Subgroup_7; f__uncultured_bacterium_o_Subgroup_7; g__uncultured_bacterium_o_Subgroup_7;	4	7
342	k__Bacteria; p__Firmicutes; c__Erysipelotrichia; o__Erysipelotrichales; f__Erysipelotrichaceae; g__Ileibacterium;	4	7
343	k__Bacteria; p__Gemmatimonadetes; c__Gemmatimonadetes; o__Gemmatimonadales; f__Gemmatimonadaceae; g__Gemmatimonas;	5	7
344	k__Bacteria; p__Proteobacteria; c__Alphaproteobacteria; o__Reyranellales; f__Reyranellaceae; g__Reyranella;	5	7
345	k__Bacteria; p__Proteobacteria; c__Gammaproteobacteria; o__Betaproteobacteriales; f__Rhodocyclaceae; g__Thauera;	5	7
346	k__Bacteria; p__Proteobacteria; c__Gammaproteobacteria; o__Betaproteobacteriales; f__Burkholderiaceae; g__Hydrogenophaga;	8	7
347	k__Bacteria; p__Proteobacteria; c__Alphaproteobacteria; o__Acetobacterales; f__Acetobacteraceae; g__uncultured_bacterium_f_Acetobacteraceae;	9	7
348	k__Bacteria; p__Proteobacteria; c__Alphaproteobacteria; o__uncultured_bacterium_c_Alphaproteobacteria; f__uncultured_bacterium_c_Alphaproteobacteria; g__uncultured_bacterium_c_Alphaproteobacteria;	11	7
349	k__Bacteria; p__Actinobacteria; c__Actinobacteria; o__Streptomycetales; f__Streptomycetaceae; g__uncultured_bacterium_f_Streptomycetaceae;	1	8
350	k__Bacteria; p__Proteobacteria; c__Alphaproteobacteria; o__Rhizobiales; f__Xanthobacteraceae; g__Pseudolabrys;	3	8
351	k__Bacteria; p__Acidobacteria; c__Acidobacteriia; o__Solibacterales; f__Solibacteraceae_Subgroup_3; g__Bryobacter;	4	8

352	k__Bacteria; p__Proteobacteria; c__Gammaproteobacteria; o__Steroidobacterales; f__Steroidobacteraceae; g__uncultured_bacterium_f_Steroidobacteraceae;	4	8
353	k__Bacteria; p__Actinobacteria; c__Actinobacteria; o__Propionibacteriales; f__Propionibacteriaceae; g__Cutibacterium;	6	8
354	k__Bacteria; p__Proteobacteria; c__Gammaproteobacteria; o__Betaproteobacteriales; f__Burkholderiaceae; g__Burkholderia-Caballeronia-Paraburkholderia;	9	8
355	k__Bacteria; p__Proteobacteria; c__Alphaproteobacteria; o__Elsterales; f__uncultured_bacterium_o_Elsterales; g__uncultured_bacterium_o_Elsterales;	10	8
356	k__Bacteria; p__Firmicutes; c__Clostridia; o__Clostridiales; f__Peptococcaceae; g__uncultured_bacterium_f_Peptococcaceae;	1290	8
357	k__Bacteria; p__Proteobacteria; c__Gammaproteobacteria; o__Betaproteobacteriales; f__Neisseriaceae; g__Neisseria;	1	9
358	k__Bacteria; p__Actinobacteria; c__Actinobacteria; o__Frankiales; f__Acidothermaceae; g__Acidothermus;	4	9
359	k__Bacteria; p__Proteobacteria; c__Gammaproteobacteria; o__Betaproteobacteriales; f__Burkholderiaceae; g__Massilia;	4	9
360	k__Bacteria; p__Actinobacteria; c__Actinobacteria; o__Micrococcales; f__Intrasporangiaceae; g__uncultured_bacterium_f_Intrasporangiaceae;	5	9
361	k__Bacteria; p__Firmicutes; c__Negativicutes; o__Selenomonadales; f__Veillonellaceae; g__Mitsuokella;	8	9
362	k__Bacteria; p__Actinobacteria; c__Actinobacteria; o__Micrococcales; f__Microbacteriaceae; g__uncultured_bacterium_f_Microbacteriaceae;	9	9
363	k__Bacteria; p__Proteobacteria; c__Gammaproteobacteria; o__Betaproteobacteriales; f__Burkholderiaceae; g__Comamonas;	10	9
364	k__Bacteria; p__Proteobacteria; c__Deltaproteobacteria; o__Desulfovibrionales; f__Desulfovibrionaceae; g__uncultured_bacterium_f_Desulfovibrionaceae;	101	9

365	k__Bacteria; p__Bacteroidetes; c__Bacteroidia; o__Bacteroidales; f__Prevotellaceae; g__Prevotellaceae_UCG-001;	1626	9
366	k__Bacteria; p__Firmicutes; c__Clostridia; o__Clostridiales; f__Family_XI; g__Anaerococcus;	3	10
367	k__Bacteria; p__Proteobacteria; c__Alphaproteobacteria; o__Sphingomonadales; f__Sphingomonadaceae; g__uncultured_bacterium_f_Sphingomonadaceae;	8	10
368	k__Bacteria; p__Proteobacteria; c__Gammaproteobacteria; o__Betaproteobacteriales; f__Nitrosomonadaceae; g__MND1;	18	10
369	k__Bacteria; p__Firmicutes; c__Clostridia; o__Clostridiales; f__Lachnospiraceae; g__[Ruminococcus]_gnavus_group;	57	10
370	k__Bacteria; p__Firmicutes; c__Clostridia; o__Clostridiales; f__Lachnospiraceae; g__Lachnospiraceae_ND3007_group;	232	10
371	k__Bacteria; p__Firmicutes; c__Clostridia; o__Clostridiales; f__Ruminococcaceae; g__Ruminiclostridium_5;	311	10
372	k__Bacteria; p__Acidobacteria; c__Acidobacteriia; o__Acidobacteriales; f__Koribacteraceae; g__Candidatus_Koribacter;	9	11
373	k__Bacteria; p__Rokubacteria; c__NC10; o__Rokubacteriales; f__uncultured_bacterium_o_Rokubacteriales; g__uncultured_bacterium_o_Rokubacteriales;	12	12
374	k__Bacteria; p__Cyanobacteria; c__Melainabacteria; o__Obscuribacterales; f__uncultured_bacterium_o_Obscuribacterales; g__uncultured_bacterium_o_Obscuribacterales;	13	12
375	k__Bacteria; p__Proteobacteria; c__Alphaproteobacteria; o__Sphingomonadales; f__Sphingomonadaceae; g__Porphyrobacter;	15	12
376	k__Bacteria; p__Acidobacteria; c__Acidobacteriia; o__Solibacterales; f__Solibacteraceae_Subgroup_3; g__Candidatus_Solibacter;	16	12

377	k__Bacteria; p__Bacteroidetes; c__Bacteroidia; o__Bacteroidales; f__Tannerellaceae; g__Parabacteroides;	923	12
378	k__Bacteria; p__Proteobacteria; c__Gammaproteobacteria; o__Alteromonadales; f__Idiomarinaceae; g__Aliidiomarina;	1	13
379	k__Bacteria; p__Proteobacteria; c__Alphaproteobacteria; o__Rhizobiales; f__Rhizobiaceae; g__Mesorhizobium;	6	13
380	k__Bacteria; p__Proteobacteria; c__Alphaproteobacteria; o__Caulobacterales; f__Caulobacteraceae; g__uncultured_bacterium_f_Caulobacteraceae;	12	14
381	k__Bacteria; p__Proteobacteria; c__Gammaproteobacteria; o__Betaproteobacteriales; f__Burkholderiaceae; g__Achromobacter;	12	14
382	k__Bacteria; p__Proteobacteria; c__Gammaproteobacteria; o__Betaproteobacteriales; f__Burkholderiaceae; g__Limnobacter;	13	14
383	k__Bacteria; p__Bacteroidetes; c__Bacteroidia; o__Flavobacteriales; f__Blattabacteriaceae; g__Candidatus_Sulcia;	20	14
384	k__Bacteria; p__Firmicutes; c__Erysipelotrichia; o__Erysipelotrichales; f__Erysipelotrichaceae; g__Holdemanella;	20	14
385	k__Bacteria; p__Firmicutes; c__Bacilli; o__Lactobacillales; f__Streptococcaceae; g__Lactococcus;	26	14
386	k__Bacteria; p__Actinobacteria; c__Coriobacteriia; o__Coriobacteriales; f__Atopobiaceae; g__Coriobacteriaceae_UCG-002;	75	14
387	k__Bacteria; p__Firmicutes; c__Clostridia; o__Clostridiales; f__Lachnospiraceae; g__Lachnospira;	1058	14
388	k__Bacteria; p__Actinobacteria; c__Actinobacteria; o__Pseudonocardiales; f__Pseudonocardiaceae; g__Pseudonocardia;	5	15
389	k__Bacteria; p__Proteobacteria; c__Gammaproteobacteria; o__uncultured_bacterium_c_Gammaproteobacteria;	7	15

	f__uncultured_bacterium_c_Gammaproteobacteria; g__uncultured_bacterium_c_Gammaproteobacteria;		
390	k__Bacteria; p__Proteobacteria; c__Alphaproteobacteria; o__Caulobacterales; f__Caulobacteraceae; g__Caulobacter;	12	15
391	k__Bacteria; p__Firmicutes; c__Clostridia; o__Clostridiales; f__Lachnospiraceae; g__Lachnoclostridium;	27	15
392	k__Bacteria; p__Firmicutes; c__Clostridia; o__Clostridiales; f__Ruminococcaceae; g__Ruminococcaceae_UCG-002;	27	15
393	k__Bacteria; p__Epsilonbacteraeota; c__Campylobacteria; o__Campylobacterales; f__Helicobacteraceae; g__Helicobacter;	173	15
394	k__Bacteria; p__Actinobacteria; c__Actinobacteria; o__Micrococcales; f__Micrococcaceae; g__Nesterenkonia;	5	16
395	k__Bacteria; p__Chloroflexi; c__AD3; o__uncultured_bacterium_c_AD3; f__uncultured_bacterium_c_AD3; g__uncultured_bacterium_c_AD3;	10	16
396	k__Bacteria; p__Firmicutes; c__Clostridia; o__Clostridiales; f__Ruminococcaceae; g__Subdoligranulum;	32	16
397	k__Bacteria; p__Verrucomicrobia; c__Verrucomicrobiae; o__Verrucomicrobiales; f__Akkermansiaceae; g__Akkermansia;	1665	16
398	k__Bacteria; p__Proteobacteria; c__Alphaproteobacteria; o__Rhizobiales; f__Beijerinckiaceae; g__Methylobacterium;	9	17
399	k__Bacteria; p__Firmicutes; c__Clostridia; o__Clostridiales; f__Lachnospiraceae; g__Dorea;	316	17
400	k__Bacteria; p__Proteobacteria; c__Alphaproteobacteria; o__Rickettsiales; f__Mitochondria; g__uncultured_bacterium_f_Mitochondria;	7	18
401	k__Bacteria; p__Proteobacteria; c__Alphaproteobacteria; o__Sphingomonadales; f__Sphingomonadaceae; g__Novosphingobium;	10	19

402	k__Bacteria; p__Gemmatimonadetes; c__Gemmatimonadetes; o__Gemmatimonadales; f__Gemmatimonadaceae; g__uncultured_bacterium_f_Gemmatimonadaceae;	17	19
403	k__Bacteria; p__Proteobacteria; c__Alphaproteobacteria; o__Rhizobiales; f__Xanthobacteraceae; g__Bradyrhizobium;	18	19
404	k__Bacteria; p__Firmicutes; c__Erysipelotrichia; o__Erysipelotrichales; f__Erysipelotrichaceae; g__Faecalitalea;	68	22
405	k__Bacteria; p__Actinobacteria; c__Actinobacteria; o__Bifidobacteriales; f__Bifidobacteriaceae; g__Bifidobacterium;	28	23
406	k__Bacteria; p__Proteobacteria; c__Alphaproteobacteria; o__Rhizobiales; f__Xanthobacteraceae; g__uncultured_bacterium_f_Xanthobacteraceae;	30	23
407	k__Bacteria; p__Firmicutes; c__Clostridia; o__Clostridiales; f__Christensenellaceae; g__Christensenellaceae_R-7_group;	1907	23
408	k__Bacteria; p__Firmicutes; c__Clostridia; o__Clostridiales; f__Ruminococcaceae; g__Ruminococcus_1;	2165	23
409	k__Bacteria; p__Proteobacteria; c__Gammaproteobacteria; o__Gammaproteobacteria_Incertae_Sedis; f__uncultured_bacterium_o_Gammaproteobacteria_Incertae_Sedis; g__uncultured_bacterium_o_Gammaproteobacteria_Incertae_Sedis;	13	24
410	k__Bacteria; p__Firmicutes; c__Clostridia; o__Clostridiales; f__Ruminococcaceae; g__Faecalibacterium;	38	24
411	k__Bacteria; p__Firmicutes; c__Negativicutes; o__Selenomonadales; f__Veillonellaceae; g__Dialister;	48	24
412	k__Bacteria; p__Firmicutes; c__Clostridia; o__Clostridiales; f__Ruminococcaceae; g__Ruminococcaceae_NK4A214_group;	646	25

413	k__Bacteria; p__Actinobacteria; c__Actinobacteria; o__Micrococcales; f__Micrococcaceae; g__uncultured_bacterium_f_Micrococcaceae;	24	26
414	k__Bacteria; p__Acidobacteria; c__Acidobacteriia; o__Acidobacteriales; f__uncultured_bacterium_o_Acidobacteriales; g__uncultured_bacterium_o_Acidobacteriales;	35	27
415	k__Bacteria; p__Cyanobacteria; c__Oxyphotobacteria; o__Nostocales; f__Chroococcidiopsaceae; g__Chroococcidiopsis_PCC_7203;	7	28
416	k__Bacteria; p__Bacteroidetes; c__Bacteroidia; o__Bacteroidales; f__Rikenellaceae; g__Rikenellaceae_RC9_gut_group;	2769	28
417	k__Bacteria; p__Actinobacteria; c__Actinobacteria; o__Micrococcales; f__Micrococcaceae; g__Enteractinococcus;	1	29
418	k__Bacteria; p__Proteobacteria; c__Alphaproteobacteria; o__Caulobacterales; f__Hyphomonadaceae; g__uncultured_bacterium_f_Hyphomonadaceae;	23	29
419	k__Bacteria; p__Firmicutes; c__Clostridia; o__Clostridiales; f__Lachnospiraceae; g__[Eubacterium]_xylanophilum_group;	1813	30
420	k__Bacteria; p__Firmicutes; c__Clostridia; o__Clostridiales; f__Ruminococcaceae; g__Ruminiclostridium_9;	2132	30
421	k__Bacteria; p__Firmicutes; c__Clostridia; o__Clostridiales; f__Ruminococcaceae; g__Ruminococcaceae_UCG-013;	213	33
422	k__Bacteria; p__Proteobacteria; c__Alphaproteobacteria; o__Caulobacterales; f__Caulobacteraceae; g__Brevundimonas;	16	34
423	k__Bacteria; p__Bacteroidetes; c__Bacteroidia; o__Bacteroidales; f__Prevotellaceae; g__Prevotella_9;	71	34
424	k__Bacteria; p__Firmicutes; c__Clostridia; o__Clostridiales; f__Ruminococcaceae; g__Oscillibacter;	2188	34

425	k__Bacteria; p__Actinobacteria; c__Coriobacteriia; o__Coriobacteriales; f__Eggerthellaceae; g__Enterorhabdus;	56	35
426	k__Bacteria; p__Bacteroidetes; c__Bacteroidia; o__Flavobacteriales; f__Weeksellaceae; g__Chryseobacterium;	33	38
427	k__Bacteria; p__Proteobacteria; c__Gammaproteobacteria; o__Betaproteobacteriales; f__Burkholderiaceae; g__uncultured_bacterium_f_Burkholderiaceae;	41	38
428	k__Bacteria; p__Bacteroidetes; c__Bacteroidia; o__Bacteroidales; f__Rikenellaceae; g__Alistipes;	3589	38
429	k__Bacteria; p__Firmicutes; c__Negativicutes; o__Selenomonadales; f__Veillonellaceae; g__Veillonella;	38	40
430	k__Bacteria; p__Firmicutes; c__Clostridia; o__Clostridiales; f__Ruminococcaceae; g__Ruminiclostridium_6;	3371	42
431	k__Bacteria; p__Proteobacteria; c__Alphaproteobacteria; o__Acetobacterales; f__Acetobacteraceae; g__Acetobacter;	23	43
432	k__Bacteria; p__Acidobacteria; c__Acidobacteriia; o__Subgroup_2; f__uncultured_bacterium_o_Subgroup_2; g__uncultured_bacterium_o_Subgroup_2;	48	44
433	k__Bacteria; p__Cyanobacteria; c__Oxyphotobacteria; o__Chloroplast; f__uncultured_bacterium_o_Chloroplast; g__uncultured_bacterium_o_Chloroplast;	30	45
434	k__Bacteria; p__Proteobacteria; c__Gammaproteobacteria; o__Pseudomonadales; f__Moraxellaceae; g__Enhydrobacter;	34	48
435	k__Bacteria; p__Firmicutes; c__Clostridia; o__Clostridiales; f__Lachnospiraceae; g__Fusicatenibacter;	5032	48
436	k__Bacteria; p__Proteobacteria; c__Alphaproteobacteria; o__Rhizobiales; f__Rhizobiaceae; g__Allorhizobium-Neorhizobium-Pararhizobium-Rhizobium;	35	51
437	k__Bacteria; p__Firmicutes; c__Bacilli; o__Lactobacillales; f__Carnobacteriaceae; g__Atopostipes;	2	53

438	k__Bacteria; p__Acidobacteria; c__Subgroup_6; o__uncultured_bacterium_c_Subgroup_6; f__uncultured_bacterium_c_Subgroup_6; g__uncultured_bacterium_c_Subgroup_6;	60	56
439	k__Bacteria; p__Firmicutes; c__Clostridia; o__Clostridiales; f__Ruminococcaceae; g__[Eubacterium]_coprostanoligenes_group;	4049	63
440	k__Bacteria; p__Actinobacteria; c__Actinobacteria; o__Micrococcales; f__Dermabacteraceae; g__Brachybacterium;	11	69
441	k__Bacteria; p__Firmicutes; c__Clostridia; o__Clostridiales; f__Ruminococcaceae; g__Ruminococcaceae_UCG-005;	7325	71
442	k__Bacteria; p__Firmicutes; c__Negativicutes; o__Selenomonadales; f__Veillonellaceae; g__Megasphaera;	149	74
443	k__Bacteria; p__Firmicutes; c__Erysipelotrichia; o__Erysipelotrichales; f__Erysipelotrichaceae; g__Faecalibaculum;	24	76
444	k__Bacteria; p__Actinobacteria; c__Actinobacteria; o__Corynebacteriales; f__Nocardiaceae; g__Rhodococcus;	35	77
445	k__Bacteria; p__Bacteroidetes; c__Bacteroidia; o__Bacteroidales; f__Muribaculaceae; g__Gram-negative_bacterium_cTPY-13;	8331	78
446	k__Bacteria; p__Proteobacteria; c__Gammaproteobacteria; o__Enterobacteriales; f__Enterobacteriaceae; g__Serratia;	46	79
447	k__Bacteria; p__Bacteroidetes; c__Bacteroidia; o__Bacteroidales; f__Prevotellaceae; g__Prevotellaceae_NK3B31_group;	5961	86
448	k__Bacteria; p__Actinobacteria; c__Nitriliruptoria; o__Nitriliruptorales; f__Nitriliruptoraceae; g__uncultured_bacterium_f_Nitriliruptoraceae;	41	88
449	k__Bacteria; p__Firmicutes; c__Clostridia; o__Clostridiales; f__Lachnospiraceae; g__Anaerostipes;	163	91

450	k__Bacteria; p__Proteobacteria; c__Gammaproteobacteria; o__Betaproteobacteriales; f__Burkholderiaceae; g__Parasutterella;	933	93
451	k__Bacteria; p__Firmicutes; c__Bacilli; o__Lactobacillales; f__Aerococcaceae; g__Aerococcus;	1	96
452	k__Bacteria; p__Firmicutes; c__Erysipelotrichia; o__Erysipelotrichales; f__Erysipelotrichaceae; g__Erysipelotrichaceae_UCG-003;	330	97
453	k__Bacteria; p__Proteobacteria; c__Alphaproteobacteria; o__Rhizobiales; f__Rhizobiales_Incertae_Sedis; g__Phreatobacter;	109	104
454	k__Bacteria; p__Firmicutes; c__Clostridia; o__Clostridiales; f__Ruminococcaceae; g__Ruminococcaceae_UCG-008;	1169	111
455	k__Bacteria; p__Proteobacteria; c__Alphaproteobacteria; o__Rhizobiales; f__Rhizobiaceae; g__Ochrobactrum;	72	119
456	k__Bacteria; p__Firmicutes; c__Bacilli; o__Bacillales; f__Family_XI; g__Gemella;	120	120
457	k__Bacteria; p__Firmicutes; c__Clostridia; o__Clostridiales; f__Lachnospiraceae; g__Lachnospiraceae_NK4A136_group;	7225	129
458	k__Bacteria; p__Firmicutes; c__Clostridia; o__Clostridiales; f__Ruminococcaceae; g__uncultured_bacterium_f_Ruminococcaceae;	8944	132
459	k__Bacteria; p__Firmicutes; c__Bacilli; o__Lactobacillales; f__Aerococcaceae; g__Globicatella;	2	133
460	k__Bacteria; p__Firmicutes; c__Bacilli; o__Lactobacillales; f__Enterococcaceae; g__Enterococcus;	37	139
461	k__Bacteria; p__Actinobacteria; c__Actinobacteria; o__Micrococcales; f__Brevibacteriaceae; g__Brevibacterium;	76	142
462	k__Bacteria; p__Proteobacteria; c__Alphaproteobacteria; o__Sphingomonadales; f__Sphingomonadaceae; g__Sphingomonas;	147	149

463	k__Bacteria; p__Proteobacteria; c__Gammaproteobacteria; o__Betaproteobacteriales; f__Burkholderiaceae; g__Delftia;	126	150
464	k__Bacteria; p__Bacteroidetes; c__Bacteroidia; o__Bacteroidales; f__Bacteroidaceae; g__Bacteroides;	5787	156
465	k__Bacteria; p__Firmicutes; c__Clostridia; o__Clostridiales; f__Ruminococcaceae; g__Ruminococcaceae_UCG-014;	2630	176
466	k__Bacteria; p__Firmicutes; c__Clostridia; o__Clostridiales; f__Lachnospiraceae; g__Roseburia;	7192	191
467	k__Bacteria; p__Proteobacteria; c__Deltaproteobacteria; o__Desulfovibrionales; f__Desulfovibrionaceae; g__Desulfovibrio;	3980	193
468	k__Bacteria; p__Proteobacteria; c__Gammaproteobacteria; o__Xanthomonadales; f__Xanthomonadaceae; g__Stenotrophomonas;	182	194
469	k__Bacteria; p__Firmicutes; c__Bacilli; o__Bacillales; f__Bacillaceae; g__Bacillus;	99	202
470	k__Bacteria; p__Cyanobacteria; c__Melainabacteria; o__Gastranaerophilales; f__uncultured_bacterium_o_Gastranaerophilales; g__uncultured_bacterium_o_Gastranaerophilales;	467	204
471	k__Bacteria; p__Bacteroidetes; c__Bacteroidia; o__Bacteroidales; f__Prevotellaceae; g__uncultured_bacterium_f_Prevotellaceae;	20537	207
472	k__Bacteria; p__Actinobacteria; c__Coriobacteriia; o__Coriobacteriales; f__Eggerthellaceae; g__Adlercreutzia;	150	217
473	k__Bacteria; p__Proteobacteria; c__Gammaproteobacteria; o__Enterobacteriales; f__Enterobacteriaceae; g__Escherichia-Shigella;	75	223
474	k__Bacteria; p__Actinobacteria; c__Actinobacteria; o__Corynebacteriales; f__Corynebacteriaceae; g__uncultured_bacterium_f_Corynebacteriaceae;	23	236

475	k__Bacteria; p__Actinobacteria; c__Actinobacteria; o__Corynebacteriales; f__Dietziaceae; g__Dietzia;	96	253
476	k__Bacteria; p__Proteobacteria; c__Gammaproteobacteria; o__Betaproteobacteriales; f__Burkholderiaceae; g__Ralstonia;	166	357
477	k__Bacteria; p__Proteobacteria; c__Gammaproteobacteria; o__Pseudomonadales; f__Moraxellaceae; g__Psychrobacter;	4	371
478	k__Bacteria; p__Proteobacteria; c__Gammaproteobacteria; o__Pseudomonadales; f__Pseudomonadaceae; g__Pseudomonas;	446	422
479	k__Bacteria; p__Patescibacteria; c__Saccharimonadia; o__Saccharimonadales; f__Saccharimonadaceae; g__Candidatus_Saccharimonas;	689	422
480	k__Bacteria; p__Proteobacteria; c__Gammaproteobacteria; o__Oceanospirillales; f__Halomonadaceae; g__Halomonas;	168	498
481	k__Bacteria; p__Proteobacteria; c__Gammaproteobacteria; o__Pseudomonadales; f__Moraxellaceae; g__Acinetobacter;	541	548
482	k__Bacteria; p__Firmicutes; c__Bacilli; o__Bacillales; f__Staphylococcaceae; g__Staphylococcus;	293	628
483	k__Bacteria; p__Firmicutes; c__Bacilli; o__Bacillales; f__Staphylococcaceae; g__Jeotgalicoccus;	41	665
484	k__Bacteria; p__Proteobacteria; c__Gammaproteobacteria; o__Betaproteobacteriales; f__Burkholderiaceae; g__Cupriavidus;	384	689
485	k__Bacteria; p__Actinobacteria; c__Actinobacteria; o__Actinomycetales; f__Actinomycetaceae; g__Actinomyces;	22	840
486	k__Bacteria; p__Actinobacteria; c__Actinobacteria; o__Micrococcales; f__Micrococcaceae; g__Rothia;	67	869
487	k__Bacteria; p__Firmicutes; c__Bacilli; o__Bacillales; f__Staphylococcaceae; g__Nosocomiicoccus;	25	880

488	k__Bacteria; p__Firmicutes; c__Clostridia; o__Clostridiales; f__Clostridiaceae_1; g__Clostridium_sensu_stricto_1;	332	891
489	k__Bacteria; p__Firmicutes; c__Clostridia; o__Clostridiales; f__Lachnospiraceae; g__Blautia;	4560	918
490	k__Bacteria; p__Firmicutes; c__Bacilli; o__Lactobacillales; f__Aerococcaceae; g__Facklamia;	19	994
491	k__Bacteria; p__Firmicutes; c__Clostridia; o__Clostridiales; f__Lachnospiraceae; g__uncultured_bacterium_f_Lachnospiraceae;	27534	1212
492	k__Bacteria; p__Firmicutes; c__Erysipelotrichia; o__Erysipelotrichales; f__Erysipelotrichaceae; g__Allobaculum;	979	1241
493	k__Bacteria; p__Proteobacteria; c__Gammaproteobacteria; o__Enterobacteriales; f__Enterobacteriaceae; g__uncultured_bacterium_f_Enterobacteriaceae;	493	1423
494	k__Bacteria; p__Proteobacteria; c__Gammaproteobacteria; o__Enterobacteriales; f__Enterobacteriaceae; g__Proteus;	32	1521
495	k__Bacteria; p__Bacteroidetes; c__Bacteroidia; o__Bacteroidales; f__Muribaculaceae; g__uncultured_bacterium_f_Muribaculaceae;	44404	1585
496	k__Bacteria; p__Firmicutes; c__Bacilli; o__Lactobacillales; f__Streptococcaceae; g__Streptococcus;	852	1867
497	k__Bacteria; p__Actinobacteria; c__Actinobacteria; o__Corynebacteriales; f__Corynebacteriaceae; g__Corynebacterium_1;	491	2254
498	k__Bacteria; p__Firmicutes; c__Erysipelotrichia; o__Erysipelotrichales; f__Erysipelotrichaceae; g__uncultured_bacterium_f_Erysipelotrichaceae;	448	2368
499	k__Bacteria; p__Firmicutes; c__Erysipelotrichia; o__Erysipelotrichales; f__Erysipelotrichaceae; g__Turicibacter;	2455	3303
500	k__Bacteria; p__Firmicutes; c__Erysipelotrichia; o__Erysipelotrichales; f__Erysipelotrichaceae; g__Dubosiella;	1016	5497

501	k__Bacteria; p__Proteobacteria; c__Gammaproteobacteria; o__Pasteurellales; f__Pasteurellaceae; g__Rodentibacter;	34	8214
502	k__Bacteria; p__Firmicutes; c__Clostridia; o__Clostridiales; f__Clostridiaceae_1; g__Candidatus_Arthromitus;	124	29634
503	k__Bacteria; p__Firmicutes; c__Clostridia; o__Clostridiales; f__Peptostreptococcaceae; g__Romboutsia;	7669	103056
504	k__Bacteria; p__Firmicutes; c__Bacilli; o__Lactobacillales; f__Lactobacillaceae; g__Lactobacillus;	67028	240091
