# Supplementary figures and images for: Structural and Functional Differences in Small Intestinal and Fecal Microbiota: 16S rRNA Gene Investigation in Rats
Source: Microorganisms. 2024 Aug 25;12(9):1764. doi: 10.3390/microorganisms12091764 (PMC11434385; doi:10.3390/microorganisms12091764)

Figure S1. The prevalence of ileum exclusive bacteria.

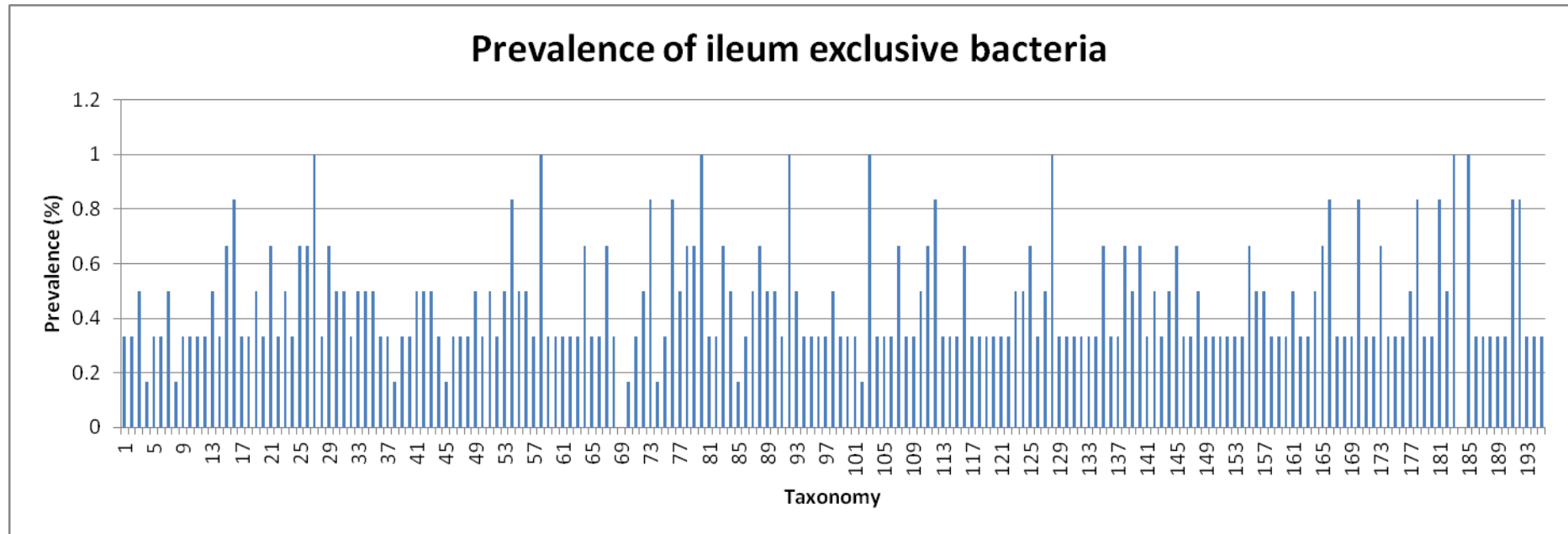

Supplement: Supplementary file 1 [file microorganisms-12-01764-s001.zip › Figure S1.pdf]

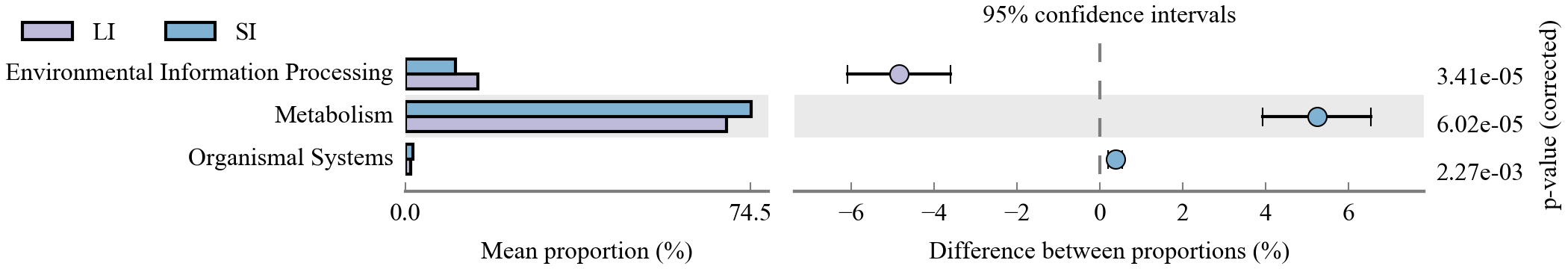

Supplement: Supplementary file 1 [file microorganisms-12-01764-s001.zip › Figure S2.png]
